# Bioactivation of cinnamic alcohol in a reconstructed human epidermis model and evaluation of sensitizing potency of the identified metabolites

**DOI:** 10.3389/ftox.2024.1398852

**Published:** 2024-07-10

**Authors:** Lorena Ndreu, Josefine Carlsson, David J. Ponting, Ida B. Niklasson, E. Johanna L. Stéen, Lukas McHugh, Niamh M. O’Boyle, Kristina Luthman, Ann-Therese Karlberg, Isabella Karlsson

**Affiliations:** ^1^ Department of Environmental Science, Exposure, and Effect, Stockholm University, Stockholm, Sweden; ^2^ Department of Materials and Environmental Chemistry, Stockholm University, Stockholm, Sweden; ^3^ Department of Chemistry and Molecular Biology, Dermatochemistry and Skin Allergy, University of Gothenburg, Gothenburg, Sweden; ^4^ Department of Chemistry and Molecular Biology, Medicinal Chemistry, University of Gothenburg, Gothenburg, Sweden; ^5^ School of Pharmacy and Pharmaceutical Sciences, Trinity College Dublin, Panoz Institute and Trinity Biomedical Sciences Institute, Dublin, Ireland

**Keywords:** cinnamic alcohol, biotransformation, reconstructed human epidermis, pOH-cinnamic alcohol, pOH-cinnamic aldehyde, cinnamic sulfate, local lymph node assay (LLNA), mass spectrometry

## Abstract

**Background:**

Cinnamic alcohol is a natural compound, widely used in fragrances, which can cause allergic contact dermatitis. Cinnamic alcohol lacks intrinsic reactivity and autoxidation or metabolic activation is necessary for it to act as a sensitizer.

**Methods:**

Bioactivation of cinnamic alcohol was explored using human liver microsomes, human liver S9 and SkinEthic™ Reconstructed Human Epidermis. A targeted multiple reaction monitoring mass spectrometry method was employed to study and quantify cinnamic alcohol along with eight potential phase I or phase II metabolites. The reconstructed human epidermis model, treated with cinnamic alcohol, was also analyzed with a non-targeted high-resolution mass spectrometry method to identify metabolites not included in the targeted method.

**Results:**

Two metabolites identified with the targeted method, namely, pOH-cinnamic alcohol and pOH-cinnamic aldehyde, have not previously been identified in a metabolic *in vitro* system. Their reactivity toward biologically relevant nucleophiles was investigated and compared to their sensitizing potency *in vivo* in the murine local lymph node assay (LLNA). According to the LLNA, the pOH-cinnamic alcohol is non-sensitizing and pOH-cinnamic aldehyde is a moderate sensitizer. This makes pOH-cinnamic aldehyde less sensitizing than cinnamic aldehyde, which has been found to be a strong sensitizer in the LLNA. This difference in sensitizing potency was supported by the reactivity experiments. Cinnamic sulfate, previously proposed as a potential reactive metabolite of cinnamic alcohol, was not detected in any of the incubations. In addition, experiments examining the reactivity of cinnamic sulfate toward a model peptide revealed no evidence of adduct formation. The only additional metabolite that could be identified with the non-targeted method was a dioxolan derivative. Whether or not this metabolite, or one of its precursors, could contribute to the sensitizing potency of cinnamic alcohol would need further investigation.

**Discussion:**

Cinnamic alcohol is one of the most common fragrance allergens and as it is more effective to patch test with the actual sensitizer than with the prohapten itself, it is important to identify metabolites with sensitizing potency. Further, improved knowledge of metabolic transformations occurring in the skin can improve prediction models for safety assessment of skin products.

## 1 Introduction

Cinnamic alcohol (CAS 104-54-1) and cinnamic aldehyde (CAS 104-55-2) are naturally found in the leaves and the inner bark of several trees from the genus *Cinnamomum*. They are also present in balms such as styrax and the *Myroxilon pereirae* resin (balsam of Peru). Cinnamic alcohol has the scent of hyacinth and is a frequent fragrance ingredient used in shampoos, soaps, fine fragrances, and other toiletries. Cinnamic aldehyde is responsible for the smell and taste of cinnamon and it is the main ingredient in the essential oil of cinnamon bark. The worldwide annual industrial usage of cinnamic aldehyde and cinnamic alcohol has been estimated to be 159 and 207 metric tons, respectively (Cocchiara et al., 2005; Letizia et al., 2005). Fragrances are common causes of contact allergy due to widespread use and frequent exposure (Diepgen et al., 2015; Bennike et al., 2017; Reeder, 2020; Amornruk et al., 2022). Some fragrance ingredients are not electrophilic and protein reactive themselves, but need to be activated first, either via autoxidation (prehapten) or bioactivation (prohapten). Cinnamic alcohol and cinnamic aldehyde are both constituents of fragrance mix I (FMI) used in the baseline series for screening of contact allergy in dermatitis patients. They are both classified as frequent contact allergens, causing allergic reactions in a notable number of persons with eczema from cosmetic products. Thus, at concentrations above 0.001% and 0.01% in leave-on products and rinse-off products, respectively, cinnamic alcohol and cinnamic aldehyde must be labeled according to the Cosmetics Directive within the European Union (SCCNFP 1999; Regulation, 2009; Uter et al., 2013).

**FIGURE 1:**
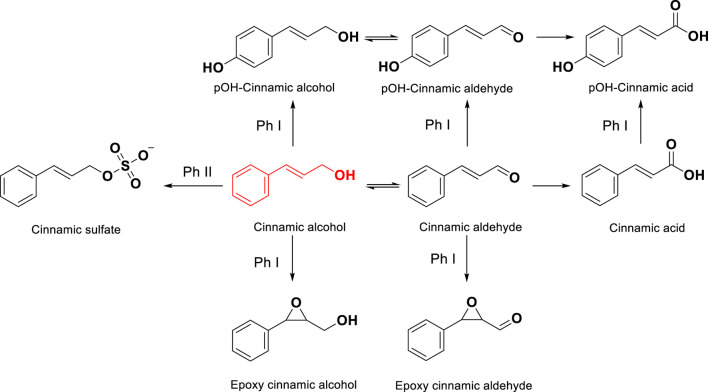
Chemical structures of cinnamic alcohol and the different metabolites expected to be formed via different phase I (Ph I) and phase II (Ph II) metabolic pathways.

The toxicological and dermatological properties of cinnamic alcohol have been extensively reviewed (Letizia et al., 2005). As cinnamic alcohol lacks structural alerts for protein reactivity, it has been shown to act as a prohapten by forming the haptens cinnamic aldehyde (Basketter, 1992; Cheung et al., 2003), epoxy cinnamic alcohol and epoxy cinnamic aldehyde (Figure 1) (Niklasson et al., 2014) via metabolic oxidation in the skin. It has also been demonstrated to be a prehapten that oxidizes rapidly upon air exposure forming cinnamic aldehyde and epoxy cinnamic alcohol (Niklasson et al., 2013). Cinnamic alcohol is frequently used as the model prohapten in mechanistic studies on skin metabolism (Basketter, 1992; Cheung et al., 1999; Smith et al., 2000; Cheung et al., 2003; Bergström et al., 2007; Ott et al., 2010).

The aim of the present study was to investigate the bioactivation of cinnamic alcohol with human liver microsomes (HLMs), human liver S9 fraction and the SkinEthic™ reconstructed human epidermis (RHE) models from EpiSkin. A sensitive targeted liquid chromatography-mass spectrometry (LC-MS) method was used to quantify the levels of cinnamic alcohol, cinnamic aldehyde and seven more potential metabolites in the three different *in vitro* systems. A non-targeted method utilizing high-resolution MS (HRMS) was used in one of the RHE experiments to detect previously unknown metabolites. Metabolites not previously detected and with suspected sensitization potency, 4-hydroxy-cinnamic alcohol (pOH-cinnamic alcohol) and 4-hydroxy-cinnamic aldehyde (pOH-cinnamic aldehyde), were further investigated regarding their reactivity toward the synthetic peptide Ac-PHCKRM, *N*-acetyl-L-cysteine (NAC) and *in silico* using methyl thiolate. These results were compared to the sensitization potential of pOH-cinnamic alcohol and pOH-cinnamic aldehyde *in vivo* in the murine local lymph node assay (LLNA) (previously unpublished results).

## 2 Material and methods

Caution: This study involves skin sensitizing compounds which should be handled with particular care.

### 2.1 Chemicals and reagents

Ac-PHCKRM was purchased from Peptide 2.0 Inc. (Chantilly, VA, United States). Corning^®^ Gentest™ Human Liver S9, Corning^®^ UltraPool™ Human Liver Microsomes, NADPH regenerating system solution A and NADPH regenerating system solution B were purchased from Corning (Corning, NY, United States). SkinEthic™ RHE models were purchased from EpiSkin (Lyon, France). Cinnamic alcohol, *trans*-cinnamaldehyde, *trans*-cinnamic acid, *trans*-cinnamic-*d*_7_ acid, pOH-cinnamic acid, Tris hydrochloride, 3′-phosphoadenosine-5′-phosphosulfate (PAPS), and tributylsulfoammonium betaine (CAS: 2364603-74-5) were purchased from Sigma-Aldrich (Steinheim, Germany). Water and acetonitrile were purchased from Fisher Scientific (Gothenburg, Sweden). Acetone was purchased from Merck (Darmstadt, Germany) and olive oil from Apoteket AB (Gothenburg, Sweden). 4-Nitrophenol, propyl 4-hydroxybenzoate, potassium dihydrogen phosphate, dipotassium hydrogen phosphate, sodium phosphate dibasic dihydrate, sodium phosphate monobasic, sodium dihydrogen phosphate monohydrate, di-sodium hydrogen phosphate dihydrate and Dulbecco’s Phosphate Buffered Saline were purchased from Merck (Darmstadt, Germany). *N*-Acetyl-L-cysteine (NAC), purity >98%, was purchased from TCI (Zwijndrecht, Belgium). 3-Phenyl-oxirane-2-yl methanol (epoxy cinnamic alcohol) was purchased from Enamine (Kyiv, Ukraine). For LC-MS standards, pOH-cinnamic alcohol was obtained from PhytoLab (Vestenbergsgreuth, Germany) and pOH-cinnamic aldehyde from AmBeed (Arlington Heights, IL, United States). For LLNA, which required larger amounts, pOH-cinnamic alcohol and pOH-cinnamic aldehyde were synthesized in house, see below. Epoxy cinnamic aldehyde was synthesized as previously described (Niklasson et al., 2013). THF used in reactions with anhydrous conditions was distilled over Na. All reactions were monitored by thin-layer chromatography (TLC) on silica plated aluminum sheets (Silica gel 60 F254, E. Merck). Spots were detected by UV light (254 or 365 nm) and anisaldehyde staining. Purification by flash column chromatography was carried out on silica gel (0.040–0.063 mm). ^1^H and ^13^C NMR spectra were measured at 400 MHz and 100 MHz, respectively. Chemical shifts are reported in ppm with the solvent residual peak as internal standard [CD_2_HOD *δ*_H_ 3.31, CD_3_OD *δ*_C_ 49.00]. All NMR experiments were measured at ambient temperature. All compounds were of purity >98% according to GC/MS before evaluation of sensitizing potential.

### 2.2 Synthesis of pOH-cinnamic alcohol

#### 2.2.1 (4-Hydroxy)cinnamic methyl ester

A 0.5 M solution of *p*-coumaric acid (2.01 g, 12.26 mmol) in MeOH (25 mL) was cooled to 0°C under nitrogen atmosphere. Thionyl chloride (0.90 mL, 12.4 mmol) was added dropwise to the solution, which was then stirred at room temperature overnight (15 h). A stream of air was bubbled through the reaction mixture for 2 h before it was concentrated under reduced pressure. The crude product was purified by flash column chromatography on silica gel with EtOAc in pentane as eluent (1:4) to afford 4-hydroxycinnamic methyl ester as a white solid (1.99 g, 91%). ^1^H NMR (400 MHz, CD_3_OD) *δ* 7.60 (d, *J* = 15.9 Hz, 1H), 7.47–7.42 (m, 2H), 6.83–6.78 (m, 2H), 6.31 (d, *J* = 15.9 Hz, 1H), 3.75 (s, 3H).^13^C NMR (100 MHz, CD_3_OD) *δ* 169.7, 161.2, 146.5, 131.1, 127.1, 116.8, 114.9, 52.0.

#### 2.2.2 (4-Hydroxy)cinnamic alcohol (pOH-cinnamic alcohol)

DIBAL (1 M in THF, 47.9 mL, 47.9 mmol) was added slowly over 15 min to a solution of 4-hydroxycinnamic methyl ester (1.99 g, 11.15 mmol) in dry THF (100 mL) at 0°C under nitrogen atmosphere. The mixture was stirred at 0°C for 1.5 h. EtOAc (20 mL) was added slowly to the mixture, followed by water (5 mL). The solvent was removed and the residue was re-dissolved in EtOAc. Water and 1 M HCl were added and the phases were separated. The aqueous phase was extracted with EtOAc. The combined organic phases were washed with brine, dried over MgSO_4_, filtered and concentrated under reduced pressure. The crude product was purified by flash column chromatography with EtOAc in pentane (1:1) as eluent to afford pOH-cinnamic alcohol (1.3 g, 79%) as a pale yellow solid. ^1^H NMR (400 MHz, CD_3_OD) *δ* 7.28–7.20 (m, 2H), 6.77–6.68 (m, 2H), 6.50 (dt, *J* = 15.8, 1.5 Hz, 1H), 6.16 (dt, *J* = 15.8, 6.0 Hz, 1H), 4.18 (dd, *J* = 6.0, 1.5 Hz, 2H). ^13^C NMR (100 MHz, CD_3_OD) *δ* 158.2, 131.9, 130.0, 128.7, 126.7, 116.3, 64.0.

### 2.3 Synthesis of pOH-cinnamic aldehyde

#### 2.3.1 4-Hydroxycinnamic aldehyde (pOH-cinnamic aldehyde)

Manganese (IV) oxide (9.45 g, 109 mmol) was added in one portion to a solution of pOH-cinnamic alcohol (1.02 g, 6.81 mmol) in dry THF (240 mL) under nitrogen atmosphere. The mixture was stirred vigorously at room temperature overnight (16 h), thereafter filtered through celite, washed with EtOAc and concentrated under reduced pressure. The crude product was purified by flash column chromatography with EtOAc in pentane (2:3) as eluent to afford the pOH-cinnamic aldehyde as a 95:5 mixture of *E*/*Z*-isomers (633 mg, 63%) as a yellow solid. ^1^H NMR (400 MHz, CD_3_OD) Minor (*Z*-isomer): *δ* 9.75 (s, 1H), 7.76 (d, *J* = 8.7 Hz, 1H), 6.93–6.89 (m, 2H). Major (*E*-isomer): *δ* 9.54 (d, *J* = 7.9 Hz, 1H), 7.62–7.34 (m, 3H), 6.88–6.79 (m, 2H), 6.59 (dd, *J* = 15.7, 7.9 Hz, 1H). ^13^C NMR (101 MHz, CD_3_OD) *δ* 196.2, 162.2, 155.9, 132.0, 127.0, 126.4, 117.0. Not all peaks are observed for the minor *Z*-isomer as they are overlapping with the major *E*-isomer.

### 2.4 Synthesis of cinnamic alcohol sulfate

#### 2.4.1 Cinnamic sulfate triethylammonium salt

Cinnamic alcohol (0.5 g, 3.73 mmol) was dissolved in dimethylformamide (DMF) (25 mL). Triethylamine (7 mL) and pyridine-SO_3_ complex (0.712 g, 1.2 eq, 4.48 mmol) were added at 0°C. The reaction was stirred overnight at room temperature. DMF was reduced under vacuum and the crude product was purified by flash column chromatography with 5% MeOH in dichloromethane to afford cinnamic alcohol sulfate triethylammonium salt (1.0 g, 85%) as a yellow oil.

#### 2.4.2 Cinnamic sulfate sodium salt

A procedure was adapted from the literature (Gill et al., 2019) as follows: Cinnamic alcohol (0.088 g, 0.66 mmol) and tributylsulfoammonium betaine (Bu_3_NSO_3_) (2.0 equiv., 0.350 g) were added to a dried flask under nitrogen. Anhydrous MeCN (1.5 mL) was added and the reaction mixture was heated to 90° for 2 h. It was then cooled to room temperature and the solvent was removed *in vacuo*. Water (10 mL) was added to the flask. The aqueous layer was extracted using ethyl acetate (4 × 10 mL). The combined organic extracts were dried over anhydrous sodium sulfate and filtered. Solvent was removed *in vacuo* to generate cinnamic sulfate tributylammonium salt, which was purified by column chromatography using silica gel (DCM:MeOH 9:1) to yield a white solid in 66% yield. ^1^H NMR (400 MHz, CDCl_3_): δ 7.23–7.40 (m, 5H), 6.66 (d, *J* = 15.9 Hz, 1H), 6.33–6.41 (m, 1H), 3.00–3.04 (m, 6H), 4.74 (d, *J* = 6.2 Hz, 2H), 1.70–1.76 (m, 6H), 1.36–1.41 (m, 6H), 0.96 (t, *J* = 7.3 Hz, 9H).

The flask containing the tributylammonium salt (0.43 mmol, 0.174 g) was charged with MeCN (5 mL) and sodium iodide (5.0 equiv., 2.2 mmol, 0.33 g). The reaction mixture was stirred vigorously for 2 h at room temperature. The white precipitated solid was washed with MeCN (2 × 10 mL) and dried to afford cinnamic sulfate sodium salt. IR. ν_max_ cm^−1^ 1,246 (O-S). MS: Found *m/z* 213.0224. ^1^H NMR (400 MHz, D_2_O): δ 7.24–7.43 (m, 5H), 6.69 (d, *J* = 15.9 Hz, 1H), 6.29–6.36 (m, 1H), 4.72 (d, *J* = 6.2 Hz, 2H). ^13^C NMR (600 MHz, D_2_O) *δ* 136.7, 130.9, 128.9, 128.2, 127.9, 126.4, 62.2.

### 2.5 Bioactivation incubations

#### 2.5.1 Liver microsomal incubations

The microsomal incubations were performed using human liver microsomes (HLMs) (0.5 mg of protein, pooled from 150 male and female donors, Corning), cinnamic alcohol (10 µM final concentration in acetonitrile), potassium phosphate buffer (100 mM, pH 7.4) and a nicotinamide adenine dinucleotide phosphate (NADPH) regenerating system (1.3 mM NADP^+^, 3.3 mM glucose-6-phosphate, 0.4 U/mL glucose-6-phosphate dehydrogenase, and 3.3 mM magnesium chloride, Corning) in a total volume of 1.0 mL. All incubations were performed in triplicate and control samples were run in the absence of the NADPH regenerating system. The incubations were initialized by the addition of cinnamic alcohol after 5 min of pre-incubation at 37°C. After 0, 10, 20, 40 and 60 min, 100 µL was withdrawn from the incubation and terminated by the addition of 100 µL of acetonitrile containing 5 µM of IS (*trans*-cinnamic-*d*_7_ acid). The extracts were collected after 3 min and centrifugated at 10,000 g, followed by LC-MS/MS analysis.

#### 2.5.2 Liver S9 fraction incubations

The S9 incubations were performed using human liver S9 (0.5 mg of protein, pooled from 20 male and female donors, Corning), cinnamic alcohol (10 µM final concentration), potassium phosphate buffer (100 mM, pH 7.4) and PAPS in a total volume of 1.0 mL. All incubations were performed in triplicate and control samples were run in the absence of PAPS. Additionally, 4-nitrophenol and propyl 4-hydroxybenzoate were used as a positive controls. The incubations were initialized by the addition of cinnamic alcohol or one of the positive controls, after 5 min of pre-incubation at 37°C. After 0, 10, 20, 40, and 60 min, 100 µL was withdrawn from the incubation and terminated by the addition of 100 µL of acetonitrile containing 5 µM of IS (*trans*-cinnamic-*d*_7_ acid). The extracts were collected after 3 min and centrifugated at 10,000 g, followed by LC-MS/MS analysis.

#### 2.5.3 SkinEthic^TM^ RHE incubations

Two sets of RHE experiments were conducted: the first set was treated with 2 µL of a 5 mM solution of cinnamic alcohol in DMSO and sampled after 2, 4, and 6 h. The second set was treated with 30 µL of a 50 mM solution of cinnamic alcohol in DMSO and sampled after 2, 4, 6, and 24 h. The incubations were performed mostly in dark to minimize autoxidation.

Upon arrival, the SkinEthic RHE model inserts were initially transferred to 6-well plates containing 1 mL of SkinEthic maintenance medium in each well. They were then pre-incubated at 37°C, 5% CO_2_, and saturated humidity for 24 h. Subsequently, the models were transferred into a 24-well plate with 300 μL of maintenance medium in each well. Next, the models were treated with cinnamic alcohol solution as described above. Control incubations were performed in the absence of cinnamic alcohol. For each sampling time point described above, all the medium was removed from the well and transferred to a vial. Following the medium removal, 300 μL of maintenance medium was added inside each insert and shaken for 30 min. Then, the same medium was added to the bottom of the insert, shaken again for 30 min, and transferred to a vial. This step allowed for the extraction of analytes that may have been retained in each insert. The incubations were performed in triplicate, and separate incubations were conducted for different time points under investigation. All samples were analyzed by LC-MS/MS analysis after addition of 5 µM of IS (*trans*-cinnamic-*d*_7_ acid).

### 2.6 Reactions toward the model peptide Ac-Pro-His-Cys-Lys-Arg-Met-OH (Ac-PHCKRM)

The experiments performed in this section were adapted from the OECD Test No. 442C: In Chemico Skin Sensitisation with some modifications (OECD, 2023). The peptide Ac-PHCKRM was prepared at 1 mM in phosphate buffer (100 mM, pH 7.5). Stock solutions (8 mM) of cinnamic alcohol, cinnamic aldehyde, pOH-cinnamic alcohol, pOH-cinnamic aldehyde and cinnamic sulfate (both cinnamic sulfate triethylammonium salt and cinnamic sulfate sodium salt) were prepared in acetonitrile. All solutions were bubbled with argon to minimize peptide dimerization. The incubation was performed in dark vials and started by addition of the peptide and test chemical at 1:10 M ratio. The final composition in each vial consisted of 25% acetonitrile and a volume of 0.5 mL. Triplicate of test chemical and peptide incubations and control samples of the peptide only were injected, and the reaction was monitored at ±1 min, 1 h, 2 h, 3 h, 4 h, 5 h. Peptide dimerization of around 10% was observed in all the peptide reactivity incubations.

### 2.7 Reactivity toward *N*-acetyl-L-cysteine

Stock solutions of NAC, cinnamic aldehyde, pOH-cinnamic aldehyde were freshly prepared in MeOH (5 mM). The stock solutions were diluted to a concentration of 0.2 mM in phosphate buffer and the reactions were initiated by mixing NAC with cinnamic aldehyde or pOH-cinnamic aldehyde, respectively (molar ratio 1:1). Sodium phosphate buffer (100 mM, pH 7.4) and MeOH were bubbled with argon for 15 min prior to the experiments to minimize the presence of oxygen. The reactions were performed in triplicate and monitored for 5 h. Control samples of cinnamic aldehyde and pOH-cinnamic aldehyde with no NAC were analyzed and used to compare peak areas.

### 2.8 Targeted LC-MS/MS analysis of formed metabolites

Analysis of the samples from the different incubations was performed on an ACQUITY Ultra performance liquid chromatography (UPLC) H-Class PLUS System coupled to a Xevo TQ-S micro triple quadrupole mass spectrometer from Waters (Massachusetts, United States). Separation of the analytes was achieved on an ACE Excel C18-PFP column (75 × 2.1 mm internal diameter, particle size 1.7 μm, from Advanced Chromatography Technologies Ltd. (Aberdeen, Scotland). Mobile phase A consisted of 0.1% formic acid in H_2_O, and mobile phase B of 0.1% formic acid in acetonitrile and the flow rate used was 0.3 mL/min. The LC gradient was kept at 15% B for the first 2 min, followed by a linear gradient from 15% to 45% of B in 10 min, the percentage of B increased from 45% to 85% between 10 and 10.5 min, kept constant at 85% for 2 min and returned to initial conditions at 12.5 min. The column was then equilibrated for 2.5 more min, resulting in a total run time of 15 min. The column temperature was 40°C and the injection volume was 2 µL. Electrospray ionization was employed, and the polarity was switched between positive and negative mode during the run depending on the analyte, Table 1. In both cases, the desolvation temperature was set to 600°C, and the desolvation and cone gas flow were set to 1,100 L/h and 100 L/h, respectively. Ion energy 2 was set at 0.4 for positive mode and at 1.2 for the negative mode. A capillary voltage of 3.0 V was used for all analytes. MS analysis was performed in multiple reaction monitoring (MRM) mode. Two transitions and the optimized collisional energy and cone voltage were included in different retention windows for each individual analyte, Table 1; Supplementary Figure S1. The identity of chromatographic peaks was confirmed by comparison to their corresponding reference compounds.

**TABLE 1:** Exact mass (Da), ionization mode, cone voltage (V), precursor ion (*m*/*z*), retention time (Rt), transitions (*m*/*z*) and the equivalent collision energy (eV) for all the compounds studied.

Compound	Exact mass (Da)	Mode	Cone voltage (V)	Parent ion (*m*/*z*)	Rt (min)	Transitions (*m*/*z*)/Collision energy (eV)
Cinnamic alcohol	134.0731	Positive	40	117.07	7.26	115 (16)
				[M + H-H_2_O]^+^		91 (20)
Cinnamic aldehyde	132.0575	Positive	30	133.06	8.79	105 (14)
				[M + H]^+^		55 (12)
Cinnamic acid	148.0524	Positive	20	149.05	8.0	131 (10)
				[M + H]^+^		103 (18)
pOH-Cinnamic alcohol	150.068	Positive	30	133.07	2.68	105 (15)
				[M + H-H_2_O]^+^		79 (22)
pOH-Cinnamic aldehyde	148.0524	Positive	25	149.05	5.28	103 (17)
						55 (14)
pOH-Cinnamic acid	164.0473	Negative	22	163.04	3.75	119 (15)
				[M + H]^−^		93 (28)
Epoxy cinnamic alcohol	150.068	Positive	38	133.07	5.01	115 (14)
				[M + H-H_2_O]^+^		79 (18)
Epoxy cinnamic aldehyde	148.0524	Positive	28	149.05	8.15	121 (12)
				[M + H]^+^		91 (18)
Cinnamic sulfate triethylammonium salt	213.0227	Negative	20	213.02	5.36	96 (25)
				[M + H]^−^		80 (17)
Cinnamic acid-*d*_7_ (IS)	155.1072	Positive	25	156.10	8.00	137 (10)
				[M + H]^+^		109 (19)

### 2.9 Evaluation of the quantification method

The detection limit (LoD) and quantification limit (LoQ) were established by analyzing the analytes at low concentrations, with LoD and LoQ calculated at 3 and 10 times the signal-to-noise ratio, respectively. Signal refers to the average response of three replicate analyses, while noise refers to the standard deviation of the same analyses. To assess the linearity of the method, calibration curves and the equivalent residuals were plotted for each analyte. The range of the calibration curves ranged from 0.01 or 0.1 µM (depending on the LoQ of the analyte) to 10 µM. For each calibration standard and analyte, the response was calculated as the ratio between the analyte peak area and the IS peak area. To determine the accuracy of the method expressed as % relative error (%RE) and the precision of the method expressed as % relative standard deviation (%RSD), four replicates of quality control (QC) samples of cinnamic alcohol at three different concentration levels, low medium and high (0.3, 1.8, and 4.1 µM) were analyzed. To evaluate the carry-over effect, instrumental blanks were analyzed with the rest of the samples. For statistical purposes, all samples were analyzed in triplicate.

### 2.10 Stability of cinnamic sulfate

A 10 µM aqueous solution of either cinnamic sulfate triethylammonium salt or cinnamic sulfate sodium salt was prepared. The mixtures were then incubated at ambient temperature for 5 h and analyzed with HRMS, as described in Section 2.11.

### 2.11 Non-target screening LC-MS/MS analysis of formed metabolites

The media samples from the second set of the treated RHE were also subject to non-target high-resolution mass spectrometry screening. For that, a Dionex UltiMate 3,000 ultrahigh performance liquid chromatograph coupled to a Q Exactive HF Orbitrap high-resolution mass spectrometer (ThermoFisher Scientific, Waltham, MA, United States) was used. The same column and LC conditions as for the target screening were applied, but here the mass spectrometry analysis acquisition was performed in Top-10 full-MS/dd-MS2. The full-MS resolution was set to 120,000, with an AGC target of 3e^6^, a maximum injection time of 100 ms and a scan range of 50–500 *m/z*. The dd-MS2 resolution was set to 30,000, with an AGC target of 1e^5^, a maximum injection time of 50 ms, an isolation window of 0.4 *m/z* and a normalized collision energy of 30 eV. The mass spectrometer was acquiring data in positive and negative mode, separately.

Compound Discoverer 3.3 from Thermo Fisher Scientific Inc. (Waltham, MA, United States) was used for processing the data. MetID w Stats Expected and Unknown w Molecular Networks was used as workflow with default values except for in the node detect compounds where the minimum peak intensity was set to 500,000 and additional the search nodes for ChemSpider, mzCloud and mass lists were added to the workflow.

### 2.12 LC-MS/MS analysis of reactions with Ac-PHCKRM and *N*-acetyl-L-cysteine

The analysis was performed on a Dionex UltiMate 3,000 ultrahigh performance liquid chromatograph coupled to a Q Exactive HF Orbitrap high-resolution mass spectrometer (ThermoFisher Scientific, Waltham, MA, United States). The chromatographic separation was achieved on an AcclaimTM RSLC 120 C18 (2.2 µm, 120 Å, 2.1 × 150 mm, Thermo Scientific, Sunnyvale, CA, United States) column with mobile phase A consisting of 0.1% formic acid in water and mobile phase B of 0.1% formic acid in acetonitrile. The flow rate was set to 0.3 mL/min and a gradient was used for elution, starting with 5% B for 2 min, followed by an increase to 60% B at 10 min, at 10.1 min B was 95% and maintained until 12.5 min and the column was re-equilibrated with 5% B for 3 min resulting in a total run time of 15 min. The column temperature was 40°C and the injection volume was 3 µL. The mass spectrometry acquisition was performed in positive mode with Top-10 full-MS/dd-MS2. The full-MS resolution was set to 120,000, with an AGC target of 3e^6^, a maximum injection time of 100 ms and a scan range of 200–2000 *m*/*z* for Ac-PHCKRM and 70–1,050 *m/z* for *N*-acetyl-L-cysteine. The dd-MS2 resolution was set to 30,000, with an AGC target of 1e^5^, a maximum injection time of 50 ms, the isolation window 0.4 *m*/*z* and a normalized collision energy of 30 eV.

### 2.13 Computational techniques

The P450 heme active site was modeled using a methoxy radical, as described in literature (Turpeinen et al., 2006; Li et al., 2012; Delaine et al., 2014; Niklasson et al., 2014). Reactivity calculations were carried out at the B3LYP-D3/6-31+G** (Ditchfield et al., 1971; Slater and James, 1974; Becke, 1988; Stephens et al., 1994) level of theory in Jaguar (Schrodinger, 2009), part of the Schrödinger suite of programs. Structures were initially energy minimized in MacroModel (Schrodinger LLC, N. Y. Macromodel, 2009) before an LST search was undertaken in Jaguar for the transition state. Calculations were performed on workstations running CentOS 6.6.

### 2.14 Sensitization experiments in mice

#### 2.14.1 Experimental animals

Female CBA/Ca mice, 8 or 9 weeks of age, were purchased from B&K Sollentuna, Sweden. The mice were housed in “hepa” filtered air flow cages and kept on standard laboratory diet and water *ad lib*. The local ethics committee in Gothenburg approved the study.

#### 2.14.2 Skin sensitizing potency of pOH-cinnamic aldehyde and pOH-cinnamic alcohol in mice

A slightly modified version of the murine local lymph node assay (LLNA) (Gerberick et al., 2007) was used to assess the sensitization potency of pOH-cinnamic aldehyde and pOH-cinnamic alcohol. The original protocol uses 3 groups of 5 animals and a control group. In order to obtain more data points, the modified protocol, as previously published (Delaine et al., 2013), uses 5 groups with 3 animals in each and one control group with 4 animals.

The mice were treated by topical application on the dorsum of both ears with pOH-cinnamic aldehyde or pOH-cinnamic alcohol (25 µL) dissolved in acetone:olive oil (AOO) (4:1 v/v) or (16:1 v/v), respectively, or with the vehicle control. All solutions were freshly prepared for every application. Each compound was tested in five different concentrations. Treatments were performed daily for three consecutive days (0, 1, and 2). Sham treated control animals received vehicle alone. On day 5, all mice were injected intravenously via the tail vein with [^3^H-methyl]thymidine (2.0 Ci/mmol, Amersham Biosciences, United Kingdom) (20 µCi) in phosphate-buffered saline (PBS, containing 137 mM NaCl, 2.7 mM KCl and 10 mM phosphate buffer, pH 7.4) (250 µL). After 5 h the mice were sacrificed, the draining lymph nodes were excised and pooled for each group, and single cell suspensions of lymph-node cells in PBS were prepared using cell strainers (Falcon, BD labware, 70 µm pore size). Cell suspensions were washed twice with PBS, precipitated with TCA (5%) and left in the refrigerator overnight. The samples were then centrifuged, re-suspended in TCA (5%) (1 mL) and transferred to scintillation cocktail (10 mL) (EcoLume, INC. Radiochemicals, United States). The [^3^H-methyl]thymidine incorporation into DNA was measured by β-scintillation counting on Beckman LS 6000 TA Instruments. Results are expressed as mean dpm/lymph node for each experimental group and as stimulation index (SI) (Gerberick et al., 2008), i.e., test group/control group ratio. Test materials that at one or more concentrations caused an SI greater than 3 were considered to be positive in the LLNA. EC_3_ values (the estimated concentration required to induce an SI of 3) were calculated by linear interpolation.

AOO (4:1) was used as the vehicle for pOH-cinnamic aldehyde, however due to the extreme polarity and thus poor solubility of pOH-cinnamic alcohol in 4:1 AOO, 16:1 AOO was chosen as the vehicle. We have previously shown that this is a suitable vehicle in the LLNA (O’Boyle et al., 2022).

## 3 Results

### 3.1 Targeted method evaluation

The LoD and LoQ for each analyte, found in Supplementary Table S1, were obtained by their analysis at low concentrations and their values were based on a signal-to-noise ratio of 3 and 10, respectively. Among the analytes, cinnamic sulfate exhibited the highest LoQ (36.1 pg on column), and pOH-cinnamic aldehyde the lowest (1.98 pg on column). The variation in LoQ observed among different compounds, notably with cinnamic sulfate showing the highest, can be attributed to the detection method. Specifically, cinnamic sulfate was identified in negative mode, which, for certain compounds, can be less sensitive compared to the positive mode. High LoQ can pose a risk of missing the analyzed compound, especially if it is present in lower concentrations than the established LoQ. The linearity and the goodness of fit of the calibration curves for each analyte were assessed by the *R* ^2^ value and the residuals plot obtained in each case, Supplementary Table S2; Supplementary Figure S2. Regression analysis of the points of the calibration curve was performed and the intercept was shown to be non-significant. To ensure linearity, the random distribution of the residuals around zero was evaluated. In general, the residuals should be randomly scattered around zero and the *R* ^2^ value for mass spectrometry data should be > 0.99. Both requirements were fulfilled for all analytes. Quadruplicate analysis of QC samples based on cinnamic alcohol at three different levels, 0.3, 1.8 and 4.1 µM showed very low %RE and %RSD for the middle and high range concentrations, indicating high accuracy and precision of the method in those ranges. However, for the lowest range of 0.3 µM, a %RE of −25 and %RSD of 14 were obtained, indicating that the method is more susceptible to variations in lower ranges, Supplementary Table S3. No carry over was observed.

### 3.2 Stability of cinnamic sulfate

The stability of cinnamic sulfate appears to be affected by its salt counterion. When synthesized as the triethylammonium salt, approximately 70% remained after 5 h in an aqueous environment. As a sodium salt, the stability was somewhat improved and cinnamic sulfate was present in levels greater than 82% after 5 h.

### 3.3 Human liver microsomal incubations

Human liver microsomes were used to study the bioactivation of cinnamic alcohol. This model system was used since similar metabolic processes, although with different kinetics, are considered to occur in the liver as in the skin (Merk et al., 2004).

**FIGURE 2:**
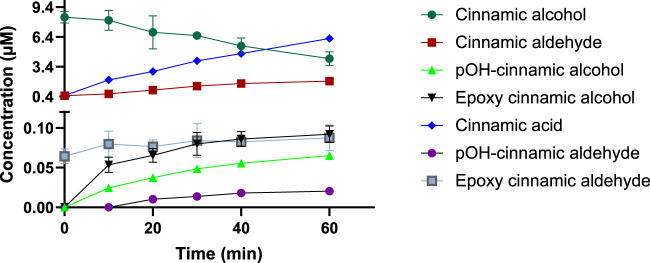
Levels (0–10 µM) of cinnamic alcohol and all its metabolites formed during HLM incubations after 10, 20, 30, 40, and 60 min (n = 3).

Six different metabolites were detected after incubation of cinnamic alcohol with the HLMs, namely, cinnamic aldehyde, pOH-cinnamic alcohol, pOH-cinnamic aldehyde, epoxy cinnamic alcohol, epoxy cinnamic aldehyde and cinnamic acid. In Figure 2, the decrease of cinnamic alcohol with time as it is being metabolized can be seen. The metabolites cinnamic acid and cinnamic aldehyde are formed in higher amounts and their concentrations are seen increasing with time. The rest of the compounds are formed at very low levels, close to their LoQ; however, an increase with increased incubation time can also be observed. More specifically, the concentration of cinnamic aldehyde ranged from 0.5 to 2 µM and cinnamic acid from 0.5 to approximately 6 µM after 60 min of incubation. From the metabolites formed at lower levels, pOH-cinnamic alcohol ranged from 0.03 to 0.07 µM, epoxy cinnamic alcohol from 0.05 to 0.1 µM, and epoxy cinnamic aldehyde from 0.07 to 0.09 µM. pOH-Cinnamic aldehyde was below LoQ in the first two time points sampled and ranged from 0.01 to 0.02 µM between 20 and 60 min. Out of all the metabolites generated, only epoxy cinnamic aldehyde did not show a significant increase over time. The levels of this metabolite detected after 60 min were comparable to those detected at the beginning of the incubation. Epoxy cinnamic aldehyde can be formed from cinnamic aldehyde but not from epoxy cinnamic alcohol (Niklasson et al., 2014). As cinnamic aldehyde is present at 0.4 μM at the beginning of the incubation it is not surprising that epoxy cinnamic aldehyde can be detected already at the first time-point. Epoxy cinnamic aldehyde has previously been shown to be unstable over time in microsomal incubations (Niklasson et al., 2014), which would explain why no increase of the metabolite is seen with time, despite availability of cinnamic aldehyde.

### 3.4 Liver S9 fraction incubations

Liver S9 incubations were conducted to investigate the potential formation of the cinnamic sulfate metabolite from cinnamic alcohol, by sulfotransferase (SULT) enzymes. In these incubations only the co-factor for SULT (PAPS) was added and not the co-factors for CYP and UGT; hence, only SULT was active. The activity of the S9 system was confirmed by the metabolism of the positive controls, 4-nitrophenol and propyl 4-hydroxybenzoate to their equivalent sulfates, Supplementary Figures S3, S4. However, cinnamic sulfate was not detected at any of the incubation time points. It is possible that this metabolite is formed but is not detected due to hydrolysis of the sulfate group in cinnamic sulfate as soon as it forms, reforming cinnamic alcohol.

### 3.5 SkinEthic™ RHE incubations

**FIGURE 3:**
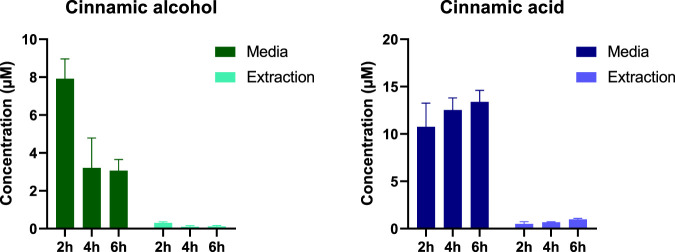
Levels of cinnamic alcohol and the only metabolite formed, namely, cinnamic acid, during the first treatment of the RHE models with 2 µL of a 5 mM cinnamic alcohol solution (n = 3).

Given that RHE models have been shown to possess metabolic enzymes that are quite similar to those found in normal human epidermis (Netzlaff et al., 2005), utilizing RHE models may be a useful approach for investigating the bioactivation of cinnamic alcohol. Therefore, two different sets of RHE experiments were performed in this study. The only metabolite detected after treatment of the RHE with a low concentration (2 µL of a 5 mM solution) of cinnamic alcohol was cinnamic acid, Figure 3. No additional metabolites could be detected after extraction of the epidermis. Although the levels of cinnamic alcohol are clearly seen decreasing with time, the levels of cinnamic acid formed are not increasing significantly. This trend, in addition to no other metabolites being detected can be an indication that potentially additional metabolites are formed but they might be reacting with proteins in the RHE models and thereafter are not migrating to the media, alternatively other metabolites are formed than those detected by the targeted method.

**FIGURE 4:**
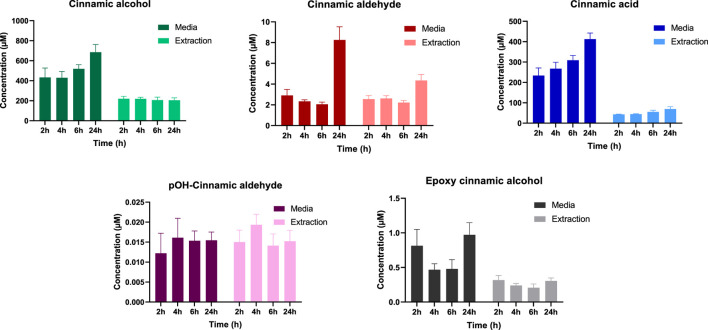
Detected levels of cinnamic alcohol and four metabolites (cinnamic aldehyde, cinnamic acid, pOH-cinnamic aldehyde and epoxy cinnamic alcohol) using a targeted LC-MS approach. The RHE models were treated with 30 µL of a 50 mM cinnamic alcohol solution (n = 3).

An approach to overcome this was to treat the RHE models with higher levels of cinnamic alcohol and follow the bioactivation for an extended time period. Hence, a second set of RHE models were treated with 30 µL of a 50 mM solution of cinnamic alcohol and sampled after 2, 4, 6, and 24 h. As seen in Figure 4, four different metabolites could be detected at the different time points. After 24 h, levels for cinnamic alcohol, cinnamic aldehyde, cinnamic acid and epoxy cinnamic alcohol could be seen increasing in the media. For pOH-cinnamic aldehyde, stable levels were observed at all time points. While the levels of the detected compounds were generally lower in the extractions of the RHE models compared to the media, in the case of pOH-cinnamic aldehyde, the same levels were present in the two sample types.

Based on the concentrations shown in Figure 4, the overall recovery of the RHE experiment, including both the added hapten and the formed metabolites, is less than 30%. This low recovery can be partly attributed to the mild extraction procedure used. Additionally, several other factors may contribute, such as the binding of more reactive metabolites to macromolecules present in the models and the formation of metabolites not included in the targeted screening. Therefore, a non-target LC-MS/MS screening of the formed metabolites in RHE media was performed.

### 3.6 Non-targeted LC-MS/MS analysis of formed metabolites in RHE

The media obtained from the second set of treated RHE samples also underwent a non-targeted screening process, with the dual purpose of a) confirming the identity of the compounds within the scope of the targeted analysis, and b) screening for potentially unidentified metabolites that were not included in the original targeted approach. The data acquired through this screening were consistent with the results of the targeted analysis, confirming the presence of cinnamic alcohol, cinnamic aldehyde, cinnamic acid, and epoxy cinnamic alcohol. However, the untargeted approach was unable to detect pOH-cinnamic aldehyde, which might be attributed to the low levels of this particular metabolite and the generally reduced sensitivity of non-targeted methods when compared to their targeted counterparts. The cinnamic sulfate metabolite could not be identified using the untargeted approach, i.e., confirming the results from the targeted analysis.

**FIGURE 5:**
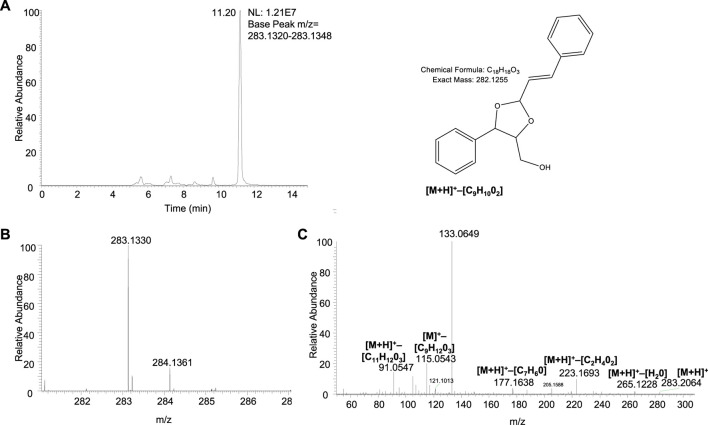
Structure, chromatogram and MS spectra of the dioxolan derivative identified in the non-targeted screening. The identification was based on: **(A)** Extracted ion chromatogram of *m*/*z* 283.1334 (mass tolerance 5 ppm) obtained from incubated RHE medium samples (24 h). **(B)** The full MS spectrum shows a measured *m*/*z* of 283.1330, with a mass difference of 1.6 ppm from the theoretical *m*/*z*. **(C)** MS/MS and a suggested fragmentation pattern further support the identity of the compound (suggested fragment structures in Supplementary Table S4).

Interestingly, the non-targeted approach suggested the presence of an additional metabolite, namely, a dioxolan derivative (Figure 5). A similar dioxolan together with a dioxolan hydroperoxide have previously been reported in the context of contact allergy as autoxidation products of geranial (Hagvall et al., 2011). We did search the non-targeted data for a dioxolan hydroperoxide, similar to that found in the work by Hagvall et al. (2011), and a compound with the correct accurate mass and a fragmentation pattern was found, which would suggest that it is a cinnamic alcohol derivative (Supplementary Figure S5; Supplementary Table S4). However, as the peak is small there is an uncertainty in the MS/MS fragmentation; therefore, we cannot confirm that this compound is indeed the dioxolan hydroperoxide of cinnamic alcohol without a synthetic standard.

Additionally, specific scans for diagnostic fragments of glutathione (308.0911, 179.0485, 233.0591 *m/z*) in the untargeted data revealed the presence of two glutathione adducts, Supplementary Figures S7, S8 with the addition of C_9_H_11_O_2_ and C_9_H_11_O to glutathione. Notably, these adducts, also contain fragments corresponding to the cinnamic alcohol derivatives (133.0648, 117.0699, 105.0700, 91.0546, *m/z*), indicating that they are likely metabolites of cinnamic alcohol bound to glutathione. These glutathione conjugates align with the different pathways described by Charpentier et al. (2018) on how cinnamic alcohol can act as a prohapten and probably correspond to an S_N_2 reaction with epoxy cinnamic alcohol to give the addition of C_9_H_11_O_2_ (Supplementary Figure S7; Supplementary Table S5) and a Michael addition to cinnamic aldehyde followed by a reduction to give the addition of C_9_H_11_O (Supplementary Figure S8; Supplementary Table S6) (Charpentier et al., 2018). No glutathione adduct corresponding to an adduct with cinnamic sulfate (addition of C_9_H_9_, *m/z* = 424.1537) was detected.

### 3.7 Computational techniques

**FIGURE 6:**
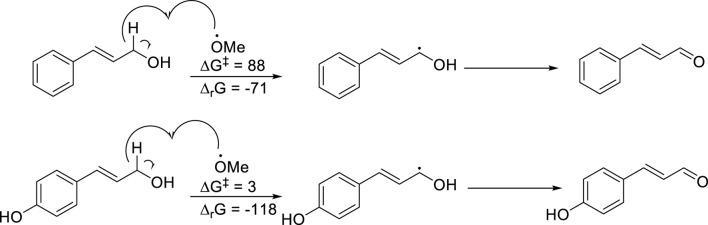
Reactivity parameters (∆G^ǂ^ and ∆_r_G) for the formation of the delocalized cinnamic radical in the case of both cinnamic alcohol and pOH-cinnamic aldehyde. All energies in kJ mol^−1^.

To compare the reactivity of cinnamic alcohol and pOH-cinnamic alcohol we modelled the P450 heme active site using a methoxy radical. In this way we could calculate the energy needed for formation of radicals from the two compounds (Figure 6). As shown, pOH-cinnamic alcohol is considerably more reactive than cinnamic alcohol and also forms a more stable radical. This may be due to the influence of the pOH group, stabilizing the radical and favouring hydrogen abstraction via a mechanism comparable to the extended conjugation in the aldehyde-quinone methide intermediate. The radical can react with other radicals, e.g., hydroxy or hydroperoxy radicals, which then can lead to the formation of the corresponding aldehydes.

**FIGURE 7:**
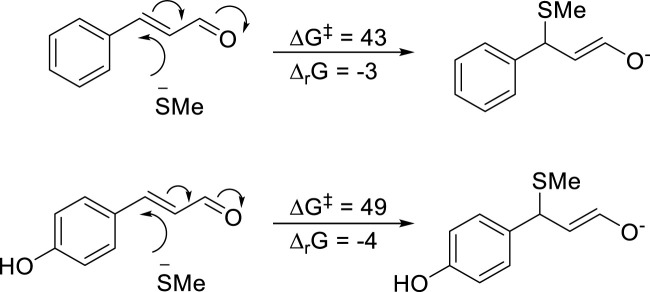
Reactivity parameters (∆G^ǂ^ and ∆_r_G) for the reaction with a model nucleophile (MeS^−^) toward both cinnamic aldehyde and pOH-cinnamic aldehyde. All energies in kJ mol^−1^.

The reactivity of the aldehydes was also compared using MeS^−^ as a model nucleophile. As shown in Figure 7, the reactivity is similar for the two analogues, with cinnamic aldehyde being somewhat more reactive.

**FIGURE 8:**
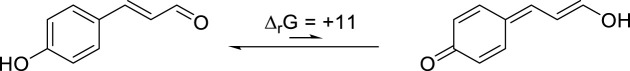
The equilibrium between pOH-cinnamic aldehyde and the possible quinone methide. Energy in kJ mol^−1^.

We also investigated the equilibrium between pOH-cinnamic aldehyde and the corresponding para-quinone methide form (Figure 8). Such quinone methides have been shown to be highly reactive, e.g., the reactive metabolite from isoeugenol (Bertrand et al., 1997). However, the equilibrium studied here was found to be directed toward the reduced form (∆_r_G = +11 kJ mol^−1^). Thus, although pOH-cinnamic alcohol is more reactive than cinnamic alcohol in radical reactions leading to formation of the corresponding aldehydes (Figure 6), the pOH-cinnamic aldehyde is less reactive toward nucleophilic groups in proteins (Figure 7). The latter data are in agreement with what is observed in the NAC depletion experiments (Figure 10).

### 3.8 Reactivity of metabolites toward model nucleophiles

**FIGURE 9:**
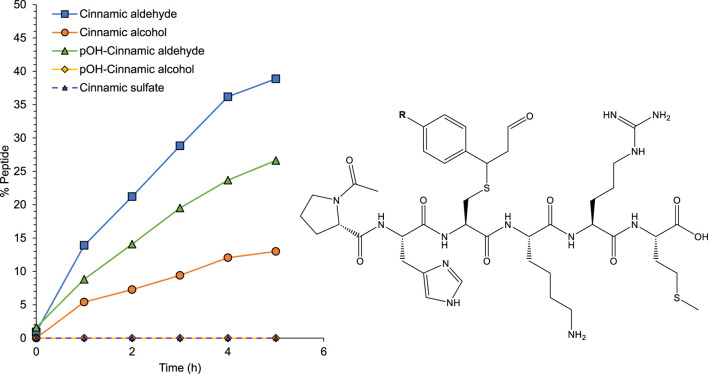
Left: Adduct formation during the reaction of cinnamic aldehyde (blue squares), cinnamic alcohol (orange circles) and pOH-cinnamic aldehyde (green triangles) with the model peptide Ac-PHCKRM. Right: The structures of the formed adducts, where R = H for cinnamic aldehyde and cinnamic alcohol, and R = OH for pOH-cinnamic aldehyde. No adduct formation was observed for pOH-cinnamic alcohol (yellow squares) or either of the cinnamic sulfate salts (purple triangle with dotted line).

A key event in both sensitization and elicitation of contact allergy is the formation of immunogenic hapten-protein complexes. Therefore, the ability of pOH-cinnamic alcohol and pOH-cinnamic aldehyde to covalently modify any of the nucleophilic amino acids within the model peptide Ac-PHCKRM was investigated. Cinnamic alcohol and cinnamic aldehyde were also incubated with the same peptide as references. The structure of the detected conjugate formed, corresponding to the Michael addition to the α,β-unsaturated bond of cinnamic aldehyde, is shown in Figure 9. After 5 h, approximately 39% of the formed adduct could be observed. Interestingly the same adduct was observed when the model peptide was incubated with cinnamic alcohol, in agreement with previously published research supporting that, in addition to being a prohapten, cinnamic alcohol is also a prehapten and oxidizes rapidly upon air exposure (Niklasson et al., 2013). As expected, the adduct formation rate was lower in this case, corresponding to approximately 13% in 5 h.

Reaction of pOH-cinnamic aldehyde with the model peptide Ac-PHCKRM led to the formation of a conjugate, also corresponding to the Michael addition to the α,β-unsaturated bond as shown in Figure 9. pOH-Cinnamic aldehyde led to an adduct formation of approximately 27%, which is lower than that of cinnamic aldehyde but higher than that of cinnamic alcohol. Incubation of the model peptide with pOH-cinnamic alcohol did not lead to any adduct formation. Incubation of the same peptide with either of the sulfate salts did not result in any adduct formation.

**FIGURE 10:**
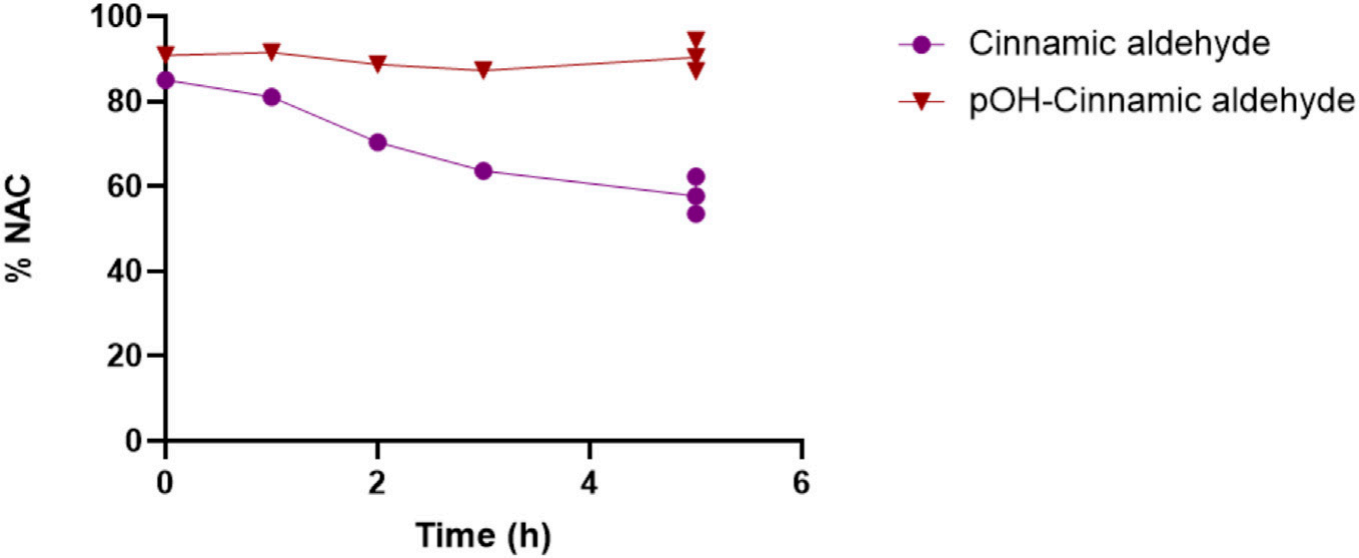
NAC depletion during the reaction with cinnamic aldehyde and pOH-cinnamic aldehyde.

The incubation of cinnamic aldehyde and its pOH equivalent with NAC resulted in a more pronounced depletion of NAC when incubated with cinnamic aldehyde, as shown in Figure 10. This observation indicates that cinnamic aldehyde is more reactive toward thiols than pOH-cinnamic aldehyde. This finding is supported by the *in silico* reactivity study with the model nucleophile MeS^−^ (Figure 7), which showed that cinnamic aldehyde is more reactive toward the model thiolate than pOH-cinnamic aldehyde.

### 3.9 Skin sensitizing potency studies

**FIGURE 11:**
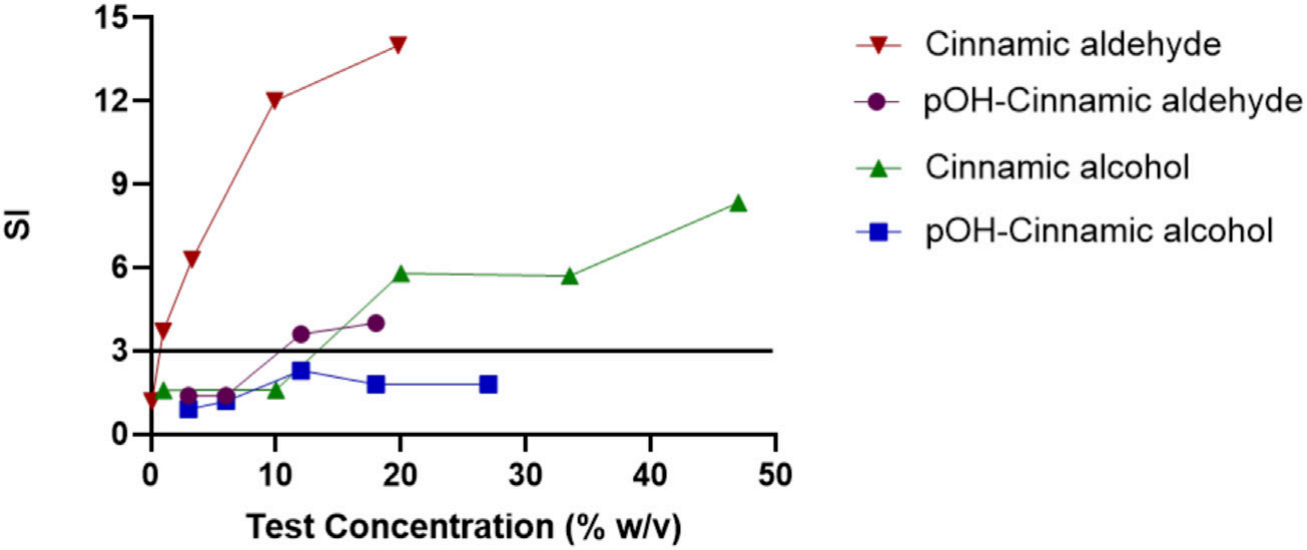
Dose-response curves in the LLNA for cinnamic aldehyde, pOH-cinnamic alcohol, cinnamic alcohol, and pOH-cinnamic aldehyde. The horizontal line marks a stimulation index (SI) of 3, the cutoff limit for a compound to be considered a sensitizer, according to the method (Gerberick et al., 2008).

Since the ability of pOH-cinnamic aldehyde to form protein conjugates was demonstrated via the reaction with the model peptide Ac-PHCKRM, we compared these results to previously unpublished results of the skin sensitizing potency of the two hydroxylated metabolites *in vivo* in the LLNA. Our group has previously reported an EC_3_ value for cinnamic aldehyde of 0.75% w/v (0.057 M) (Niklasson et al., 2013). For comparison, cinnamic alcohol was also assessed in house and yielded an EC_3_ value of 13% w/v (0.99 M). pOH-Cinnamic aldehyde gave an EC_3_ value of 6.1% w/v (0.42 M) which corresponds to almost a ten-fold decrease in sensitization potency compared to cinnamic aldehyde. None of the concentrations of pOH-cinnamic alcohol evaluated resulted in an EC_3_ value (>1.8 M, 27% w/v), Supplementary Table S7; Figure 11. Based on these results, pOH-cinnamic alcohol is classified as a non-sensitizer up to 27%, while pOH-cinnamic aldehyde is a moderate sensitizer.

## 4 Discussion

Cinnamic alcohol is almost as frequent a cause of allergic contact dermatitis as cinnamic aldehyde (Uter et al., 2013), and both are included in the fragrance mix I (FMI) in the baseline series for diagnosis of contact allergy. The high frequency of allergic reactions to test preparations of cinnamic alcohol might be explained by a higher degree of exposure to cinnamic alcohol compared to that of cinnamic aldehyde (Girardin et al., 2006). Concomitant reactions to cinnamic alcohol and cinnamic aldehyde are frequently seen which is explained by co-exposure and cinnamic alcohol causing allergy due to its activation to cinnamic aldehyde (Schnuch et al., 2007; Hagvall et al., 2018). However, among consecutively tested patients, 30%–50% of those with positive reactions to either cinnamic alcohol or cinnamic aldehyde reacted only to one of the compounds (Geier and Schnuch, 1997; Schnuch et al., 2007; Geier et al., 2015). Since experimental studies have shown that cinnamic alcohol can act both as a pre- and a prohapten forming highly sensitizing epoxides (Niklasson et al., 2013; Niklasson et al., 2014) a new patch test study was performed by Hagvall et al. (2018). Epoxy cinnamic alcohol and epoxy cinnamic aldehyde were tested in 393 consecutive dermatitis patients in parallel to screening with the baseline series containing cinnamic aldehyde and cinnamic alcohol (Hagvall et al., 2018). The conclusion from this study was that screening with the new sensitizers did not identify new cases since patients reacting to the epoxides also reacted to the aldehyde or the alcohol. Thus, the identification and assessment of the sensitizing potency of other, previously unidentified metabolites, is crucial in order to fully understand the mechanisms behind the sensitizing potential of cinnamic alcohol. In this context, it is important to point out that screening of dermatitis patients for contact allergy can be inconclusive regarding weak positive reactions which are often seen for fragrance allergens. At repeated testing a negative reaction can be positive and *vice versa*, showing the importance of rigorous testing with different test concentrations. It is also important to investigate the history of the patient but as was shown in Hagvall et al. (2018) this could be misleading since individuals might not always be aware of the cause of their allergy due to ubiquitous exposure.

In the present study, bioactivation of cinnamic alcohol in three different *in vitro* systems was investigated. Incubation of cinnamic alcohol with HLMs led to the identification and quantification of previously known metabolites, such as epoxy cinnamic aldehyde, epoxy cinnamic alcohol and cinnamic acid, but also of new metabolites, namely, pOH-cinnamic alcohol and pOH-cinnamic aldehyde. The obtained results align with those of Niklasson et al. (2014), who demonstrated the formation of cinnamic aldehyde, epoxy cinnamic alcohol, epoxy cinnamic aldehyde, and cinnamic acid in similar incubations. However, in contrast to the study by Niklasson et al. (2014), which identified epoxy cinnamic alcohol as the primary metabolite, this study found it to be a minor metabolite. Instead, cinnamic acid and cinnamic aldehyde were found to be the predominant metabolites produced. The variation in results can be explained by differences in experimental and quantification techniques employed in the two studies. Niklasson et al. (2014) utilized GC/MS analysis to examine the formation of epoxy compounds after drying the HLM extracts on molecular sieves before analysis; whereas, the current study utilized a highly sensitive LC-MS/MS approach for quantification.

Biotransformation of cinnamic alcohol using the RHE models led to the formation of cinnamic aldehyde, cinnamic acid, pOH-cinnamic aldehyde and epoxy cinnamic alcohol. These findings were substantiated through the analysis of the RHE media using a non-targeted approach. There are two main studies available in which the bioactivation of cinnamic alcohol using RHE models has been investigated. The first study, conducted by Géniès et al. (2020), examined the formation of free metabolites and protein adducts after incubation of EpiSkin™ S9 subcellular fractions and *in vitro* human skin explants with 10 different compounds, including cinnamic alcohol. The authors only detected trace amounts of cinnamic acid in the RHE model, whereas multiple metabolites were detected in the skin explants, including hydroxycinnamic acid and cinnamic acid glucuronide. In a separate study, Moss et al. (2016) investigated bioactivation of cinnamic alcohol in RHE using high-resolution magic angle spinning nuclear magnetic resonance. The authors detected two metabolites (a triol and an allylic sulfide) that they suggested were further reaction products of epoxy cinnamic alcohol and cinnamic sulfate and that these two metabolites are the ones that can act as potential electrophiles and thus are the main culprits of the sensitizing potency of cinnamic alcohol. Although it has been hypothesized that cinnamic alcohol can cause skin sensitization through a separate mechanism from the pathway involving cinnamic aldehyde—for example, via the formation of epoxy cinnamic alcohol (Niklasson et al., 2013; 2014)–and should be acknowledged as a skin sensitizer in its own right, Moss et al. (2016) were the first to suggest the potential formation of cinnamic sulfate. A recent paper by Roberts used a computational model to explore the reactivity of sulfated primary alcohols relevant to skin allergy (Roberts, 2024). In a study by Charpentier et al. (2018) in which the effect of fluorination of cinnamic alcohol on skin sensitizing potential is investigated, the authors showed that cinnamic sulfate is formed in rat liver S9 incubations. However, no correlation between *in vitro* skin sensitizing potential and degree of sulfate formation could be seen, indicating that the sulfate itself is not sensitizing. In the present study, the two *in vitro* systems tested that have the potential to form cinnamic sulfate—the human liver S9 fraction and RHE models—did not lead to the detection of this metabolite at any given time point, which is consistent with previous research findings (Niklasson et al., 2014; Géniès et al., 2020). In this work, human S9 was used whereas Charpentier et al. (2018), in which cinnamic sulfate was detected, used rat S9. Several studies have compared SULT enzyme activity across different species and found significant differences, which could explain the different results (Gamage et al., 2006; Runge-Morris et al., 2013). In the current study, cinnamic aldehyde was detected at relatively high levels and was the second most abundant metabolite after cinnamic acid in both the liver microsome and the RHE experiment (with higher concentration). The search for glutathione adducts in the HRMS non-targeted analysis also revealed an adduct that is believed to be formed by reduction of the Michael adduct of glutathione and cinnamic aldehyde (Supplementary Figure S8). One more glutathione adduct could be detected that had clear diagnostic fragments corresponding to both glutathione and a cinnamic alcohol derivative (Supplementary Figure S7). This adduct is believed to be formed by conjugation of glutathione with epoxy cinnamic alcohol.

Our HRMS non-targeted analysis also revealed the presence of a dioxolan metabolite (Figure 6). Previous studies by Hagvall et al. (2011) on the autoxidation of geranial showed the formation of a similar dioxolan derivative as the one detected for cinnamic alcohol. The study by Hagvall et al. (2011) also identified a dioxolan hydroperoxide which was found to be a strong sensitizer in the LLNA with an EC_3_ value of 0.93% w/v (0.026 M). Hagvall et al. (2011) proposed two potential mechanisms for the formation of the geranial dioxolan hydroperoxide, either via general autoxidation of the dioxolan or from a reaction of the geranial epoxide with a geranial acyl radical. As they identified the dioxolan hydroperoxide in the autoxidation mixture at earlier time points than the dioxolan itself they hypothesized that the main mechanism for dioxolan hydroperoxide is by reaction of geranial epoxide with a geranial acyl radical (Hagvall et al., 2011). When searching our non-targeted data, there are peaks that could potentially be the dioxolan hydroperoxide and the dioxolanaldehyde (Supplementary Figures S5, S6) i.e., the accurate mass and fragmentation pattern matches the suggested compounds. However, due to the low levels of these two potential compounds, synthetic standards are needed to confirm the identity. The dioxolan itself is unlikely to contribute to the sensitizing potency of metabolized/oxidized cinnamic alcohol. The dioxolanaldehyde and especially the dioxolan hydroperoxide, on the other hand, are potential haptens; hence, it is important to investigate the formation of these dimeric species from cinnamic alcohol in order to fully understand the underlying mechanisms of contact allergy caused by cinnamic alcohol.

This study is the first to identify pOH-cinnamic alcohol and pOH-cinnamic aldehyde as free metabolites in biotransformation studies of cinnamic alcohol and cinnamic aldehyde. The reactivity of these two metabolites was investigated using a model synthetic peptide and NAC and in addition the two compounds were evaluated *in silico* using a model thiolate as nucleophile. These results were compared to previously generated but unpublished *in vivo* data (murine LLNA), which showed that pOH-cinnamic alcohol is a non-sensitizer up to 27% (>1.8 M), while pOH-cinnamic aldehyde was found to be a moderate sensitizer with an EC_3_ value of 6.1% (0.42 M). This makes pOH-cinnamic aldehyde into a weaker sensitizer than cinnamic aldehyde which classifies as a strong sensitizer with an EC_3_ value of 0.75% (0.057 M). The LLNA results are confirmed by both the reactivity studies toward NAC and the peptide Ac-PHCKRM, as well as by the *in silico* reactivity study using a model thiolate nucleophile, which all showed that cinnamic aldehyde is more reactive toward thiols than p-OH cinnamic aldehyde.

In conclusion, we have identified and assessed the sensitizing potency of two previously unidentified metabolites of cinnamic alcohol, namely, pOH-cinnamic alcohol and pOH-cinnamic aldehyde. We have previously shown that pOH-cinnamic aldehyde is a moderate sensitizer *in vivo* and may therefore contribute to the total sensitizing potency of cinnamic alcohol, whereas pOH-cinnamic alcohol is unlikely to do so. Cinnamic sulfate, which has previously been suggested to contribute to the allergenic potency of cinnamic alcohol, could not be detected in either the liver S9 incubation or in the RHE model. Further, cinnamic sulfate caused no peptide depletion and no adducts could be detected. Hence, we were unable to find any support for that cinnamic sulfate is contributing to the allergenic potency of cinnamic alcohol. In addition to the metabolites detected in the target analysis, the untargeted HRMS analysis did reveal the presence of a dioxolan derivative. Although it is unlikely that this compound in itself contributes to the sensitizing potency of cinnamic alcohol, it has been reported that a similar dioxolan derivative can be formed by autoxidation of geranial and the formation of that dioxolan is via a dioxolan hydroperoxide. It would therefore be of interest to study the formation of the cinnamic alcohol dioxolan in more detail to be able determine if its formation is preceded by a hydroperoxide or any other potentially sensitizing metabolite.

Cinnamic alcohol is one of the most common fragrance allergens and although many of the individuals that display a positive patch test reaction to cinnamic alcohol also react to cinnamic aldehyde, there is a large portion who do not. Natural materials such as fragrances have a complex composition with compounds that can be activated both by autoxidation (prehaptens) and metabolically (prohaptens). Cinnamic alcohol can act both as a prehapten and a prohapten and experimental investigations have shown that in many cases the same haptens are formed via both routes. It is therefore difficult to identify what compound is the most important allergen of cinnamic alcohol without further studies including autoxidation and stability experiments, metabolic transformation in the skin including detection of modified skin proteins and improved patch testing. In general, it is easier to identify a contact allergy by patch testing with the actual sensitizer instead of the prohapten; therefore, it is important to identify and assess other potentially sensitizing metabolites of cinnamic alcohol. Moreover, increased understanding of the metabolic transformations that occur in the skin can be used to improve prediction models and safety assessments of fragrance ingredients so that skin products with less risk of sensitizing and/or eliciting an allergic reaction can be developed.

## Data Availability

The original contributions presented in the study are included in the article/Supplementary Material, further inquiries can be directed to the corresponding author.
