# Supplementary material for: Bioactivation of cinnamic alcohol in a reconstructed human epidermis model and evaluation of sensitizing potency of the identified metabolites
Source: Front Toxicol. 2024 Jul 10;6:1398852. doi: 10.3389/ftox.2024.1398852 (PMC11266153; doi:10.3389/ftox.2024.1398852)
Supplement: Supplementary file 1 [file DataSheet1.pdf]

Supplementary Material for:

Bioactivation of Cinnamic Alcohol in a Reconstructed Human Epidermis Model and Evaluation of Sensitizing Potency of the Identified Metabolites

Lorena Ndreu[1], Josefine Carlsson[2], David J. Ponting[3], Ida B. Niklasson[3], E. Johanna L. Stéen[4], Lukas McHugh[1], Niamh M. O'Boyle[5], Kristina Luthman[4], Ann-Therese Karlberg[3], Isabella Karlsson[1*]

[1]Department of Environmental Science, Exposure and Effect, Stockholm University, Stockholm, Sweden

[2]Department of Materials and Environmental Chemistry, Stockholm University, Stockholm, Sweden

[3]Department of Chemistry and Molecular Biology, Dermatochemistry and Skin Allergy, University of Gothenburg, Gothenburg, Sweden

[4]Department of Chemistry and Molecular Biology, Medicinal Chemistry, University of Gothenburg, Gothenburg, Sweden

[5]School of Pharmacy and Pharmaceutical Sciences, Trinity College Dublin, Panoz Institute and Trinity Biomedical Sciences Institute, Dublin, Ireland

[*]Correspondence
Isabella.Karlsson@aces.su.se

TABLE OF CONTENTS

TARGETED LC-MS/MS ANALYSIS OF FORMED METABOLITES IN HUMAN LIVER MICROSOMES AND SKINETHIC™ RECONSTRUCTED HUMAN EPIDERMIS 3

__Figure S1.__ Compound name, parent ion and fragments used in the MRM method for the quantification of the formed metabolites. 3
__Table S1.__ Limit of detection (LoD) and limit of quantification (LoQ) for all the metabolites expressed in nM, pg/mL and pg on column. 4
__Table S2.__ Linearity assessment including range in µM, R squared, and slope values for the calibration curves of each analyte. 4
__Figure S2__. Calibration curves and the equivalent residuals plot for each compound quantified. 5
__Table S3.__ Accuracy, expressed as % relative error (%RE), and the precision of the method, expressed as % relative standard deviation (%RSD), calculated after analysis of cinnamic alcohol at three different concentration levels. 6

POSITIVE CONTROLS LIVER S9 FRACTION INCUBATIONS 7

__Figure S3.__ Positive control S9: 4-nitrophenol 7
__Figure S4.__ Positive control S9: propyl 4-hydroxybenzoate 8

NON-TARGETED LC-MS/MS ANALYSIS OF FORMED METABOLITES IN SKINETHIC™ RECONSTRUCTED HUMAN EPIDERMIS 9

__Figure S5.__ Data could suggest the presence of a dioxolan hydroperoxide in RHE incubated with cinnamic alcohol. 9
__Figure S6.__ Data could suggest the presence of a dioxolan aldehyde in RHE incubated with cinnamic alcohol. 10
__Table S4.__ Suggested fragmentation structures for the dioxolan derivate identified in the non-target screening.[b] 11
__Figure S7.__ Data suggesting the presence of an epoxy cinnamic alcohol-glutathione adduct 12
__Table S5.__ Suggested fragmentation structures for the epoxy cinnamic alcohol-glutathione adduct identified in the non-target screening.[b] 13
__Figure S8.__ Data suggesting the presence of a cinnamic aldehyde-glutathione adduct 15
__Table S6.__ Suggested fragmentation structures for the glutathione-cinnamic aldehyde adduct identified in the non-target screening.[b] 16

MURINE LOCAL LYMPH NODE ASSAY 18

__Table S7__. Results from the murine local lymph node assay (LLNA)[a]: Sensitization experiments of the two pOH-cinnamic compounds and the control cinnamic aldehyde. 18

REFERENCES 19

Targeted LC-MS/MS analysis of formed metabolites in human liver microsomes and SkinEthic™ reconstructed human epidermis

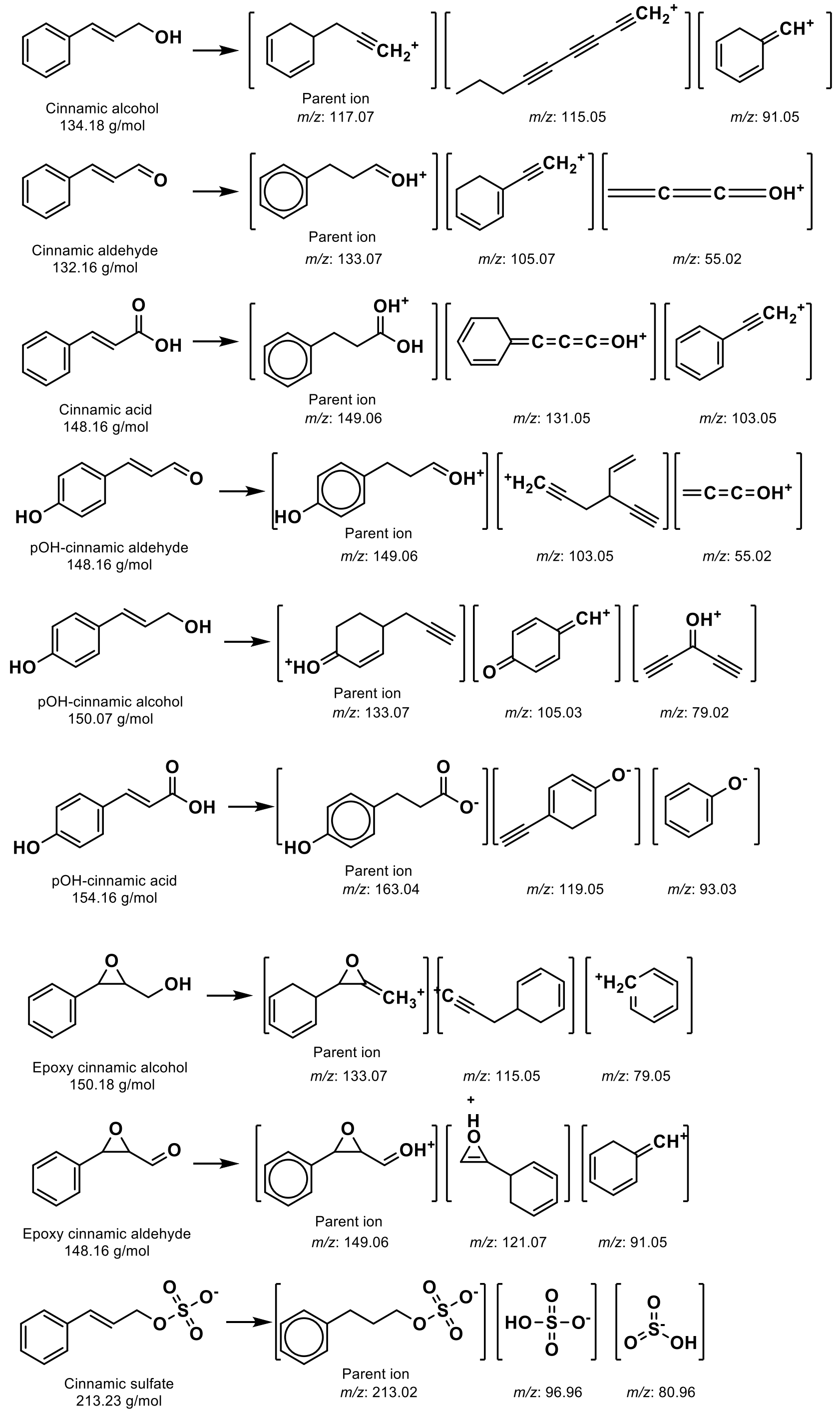

Figure S1. Compound name, parent ion and fragments used in the MRM method for the quantification of the formed metabolites.

Table S1. Limit of detection (LoD) and limit of quantification (LoQ) for all the metabolites expressed in nM, pg/mL and pg on column.

Compound	LOD (nM)	LOD (pg/µL)	LOD (pg on column)	LOQ (nM)	LOQ (pg/µL)	LOQ (pg on column)
Cinnamic alcohol	2.6	0.35	0.7	8.6	1.15	2.3
Cinnamic aldehyde	11.4	1.51	3.02	38.1	5.04	10.08
Cinnamic acid	14.8	2.19	4.38	49.3	7.30	14.6
pOH-Cinnamic alcohol	2.3	0.35	0.7	7.8	1.17	2.34
pOH-Cinnamic aldehyde	2.0	0.30	0.6	6.7	0.99	1.98
pOH-Cinnamic acid	19.9	3.27	6.54	66.4	10.90	21.8
Epoxy cinnamic alcohol	24.8	3.72	7.44	82.8	12.43	24.86
Epoxy cinnamic aldehyde	18.5	2.74	5.48	61.6	9.13	18.26
Cinnamic sulfate	25.4	5.42	10.84	84.6	18.04	36.08

Table S2. Linearity assessment including range in µM, R squared, and slope values for the calibration curves of each analyte.

Compound	Concentration range (µM)	R^2	Slope
Cinnamic alcohol	0.01-10	0.9977	0.1863
Cinnamic aldehyde	0.1-10	0.9986	0.02406
Cinnamic acid	0.1-10	0.9979	0.3049
pOH-Cinnamic alcohol	0.01-10	0.9976	0.1064
pOH-Cinnamic aldehyde	0.01-10	0.9984	0.1944
pOH-Cinnamic acid	0.1-10	0.9981	0.0004720
Epoxy cinnamic alcohol	0.5-5	0.9914	0.03447
Epoxy cinnamic aldehyde	0.1-8	0.9968	0.01875
Cinnamic sulfate	0.5-5	0.9940	0.0008440

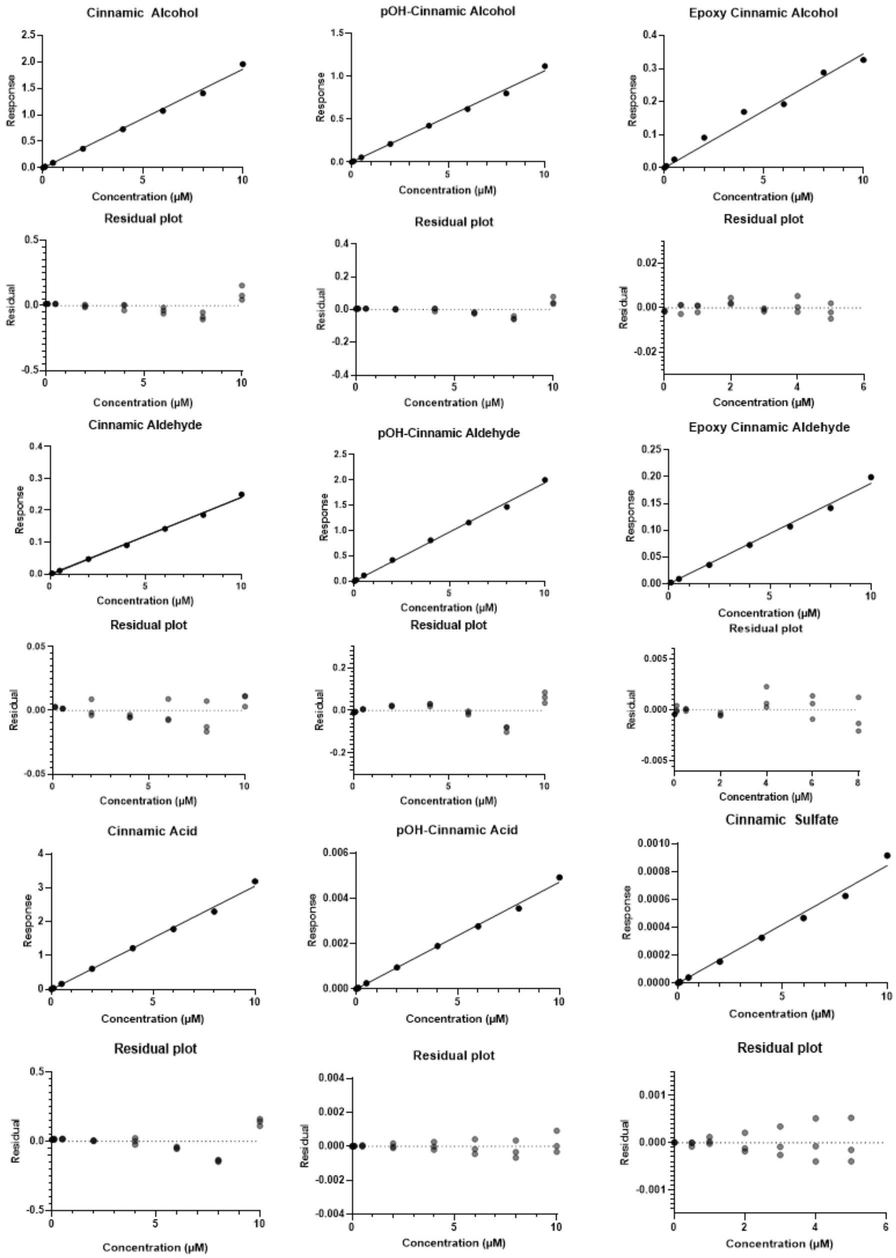

Figure S2. Calibration curves and the equivalent residuals plot for each compound quantified.

Table S3. Accuracy, expressed as % relative error (%RE), and the precision of the method, expressed as % relative standard deviation (%RSD), calculated after analysis of cinnamic alcohol at three different concentration levels.

**Sample**	**True concentration (µM)**	**Accuracy (%RE)**	**Precision (%RSD)**
QC1	0.3	-25.24	14.06
QC2	1.8	3.35	0.97
QC3	4.1	1.51	0.57

Positive controls Liver S9 fraction incubations

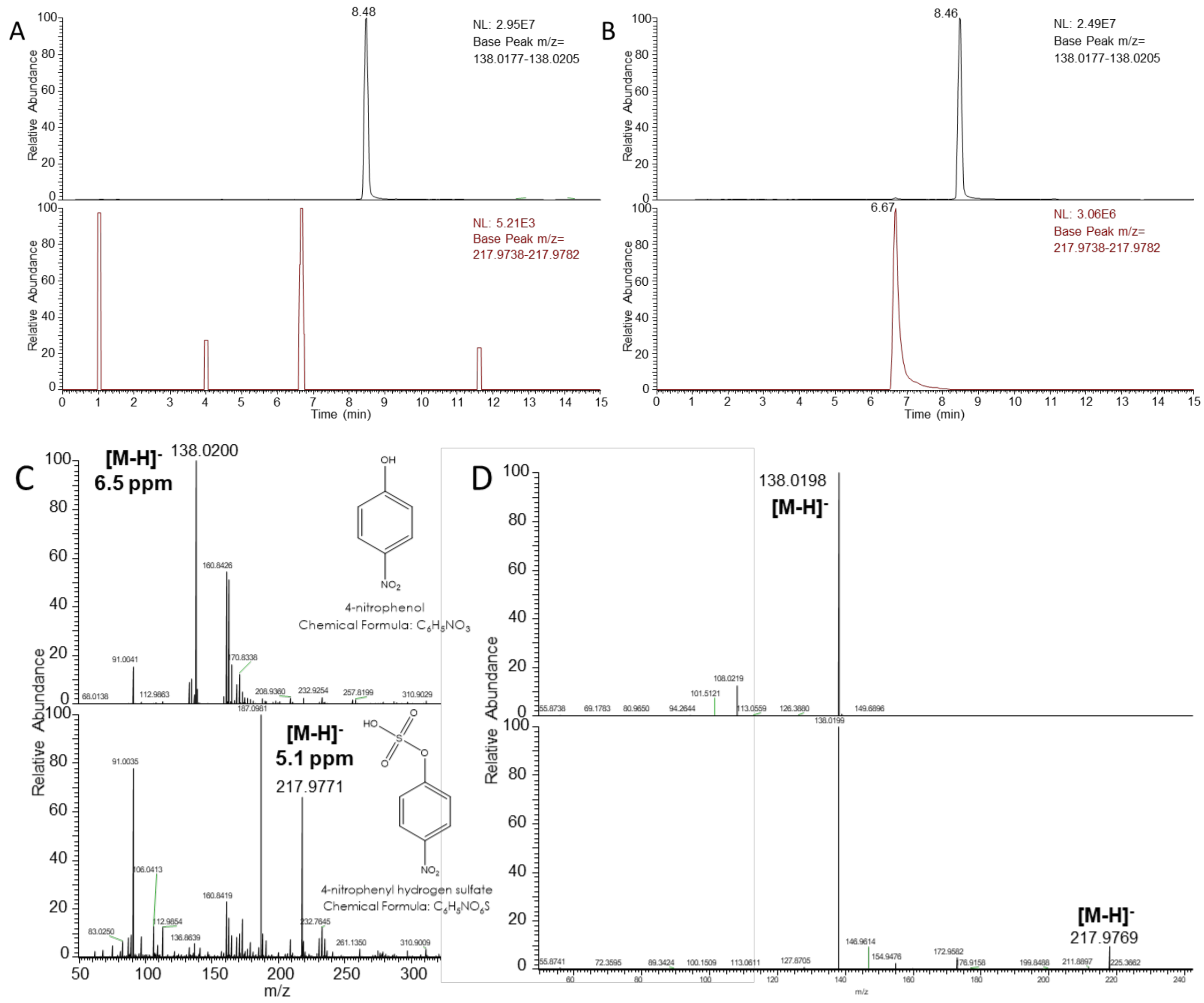

Figure S3. Positive control S9: 4-nitrophenol

A) Extracted ion chromatograms (10 ppm mass accuracy) of the positive control 4-nitrophenol in S9 incubations at time 1 min. The top chromatogram represents 4-nitrophenol (*m/z* 138.0191), while the lower chromatogram shows the corresponding sulfate ion (*m/z* 217.9759), which is absent at 1 min. **B)** Extracted ion chromatograms (10 ppm mass accuracy) after 60 minutes of S9 with PAPS incubation. The top chromatogram corresponds to 4-nitrophenol, with the area reduced to 88% compared to the 1-minute mark. The lower chromatogram represents the formed 4-nitrophenol hydrogen sulfate ion. **C)** The full MS show the measured *m/z* of 138.0200 for nitrophenol (top) and 217.9771 for the sulfate ion (bottom). The measured *m/z* values were within 7 ppm mass accuracy. **D)** The acquired MS/MS spectra of *m/z* 138.0198 (upper spectrum) and *m/z* 217.9771 (lower spectrum) correspond to 4-nitrophenol and 4-nitrophenol hydrogen sulfate ion, respectively.

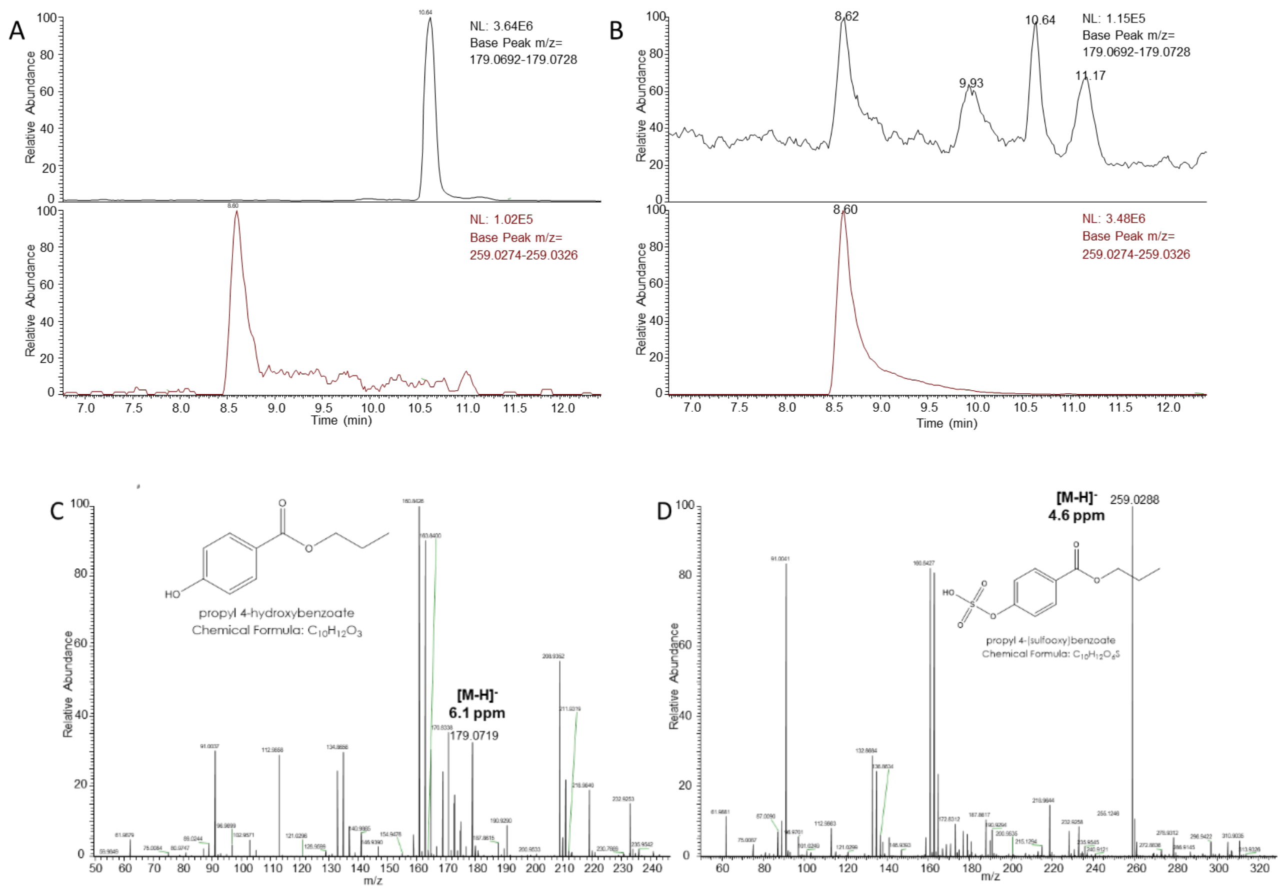

Figure S4. Positive control S9: propyl 4-hydroxybenzoate

A) Extracted ion chromatograms (10 ppm mass accuracy) of the positive control propyl 4-hydroxybenzoate in S9 incubations at time 1 min. The top chromatogram represents propyl 4-hydroxybenzoate (*m/z* 179.0708), while the lower chromatogram shows the corresponding sulfate ion (*m/z* 259.0276), which is present at low amounts already at 1 min. **B)** Extracted ion chromatograms (10 ppm mass accuracy) after 60 minutes of S9 with PAPS incubation. The top chromatogram corresponds to 4 propyl 4-hydroxybenzoate, with the area reduced to 5.2% compared to the 0-minute mark. The lower chromatogram represents the formed corresponding sulfate ion. **C)** The full MS shows the measured *m/z* of 179.0719 (mass accuracy 6.1 ppm) for 4 propyl 4-hydroxybenzoate. **D)** The full MS shows the measured *m/z* 259.0288 for the corresponding sulfate ion (mass accuracy 4.6 ppm).

Non-targeted LC-MS/MS analysis of formed metabolites in SkinEthic™ reconstructed human epidermis

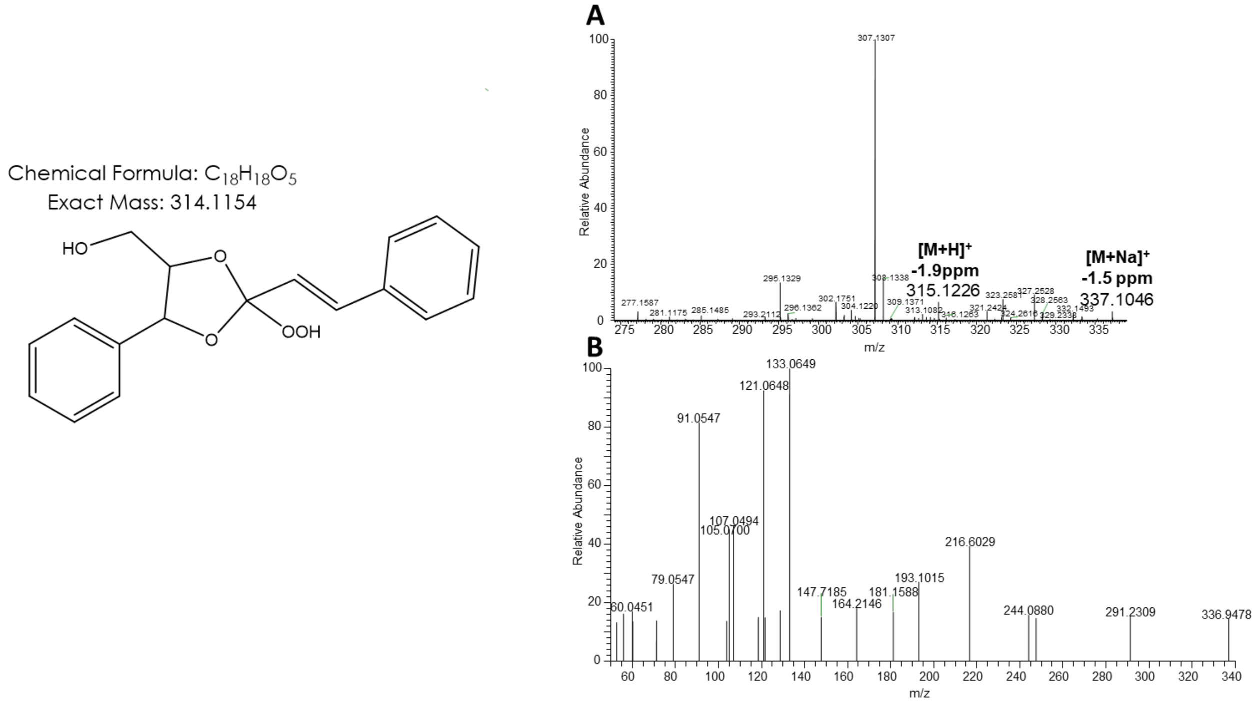

Figure S5. Data could suggest the presence of a dioxolan hydroperoxide in RHE incubated with cinnamic alcohol.

This is supported by: **A)** At RT 10.75 min, the full MS spectrum indicates: *m/z* 315.1226 and *m/z* 337.1046. These measured *m/z* values show a mass deviation of less than 2 ppm from the theoretical ions corresponding to $[M+H]^+$ and $[M+Na]^+$, respectively; **B)** The MS/MS spectrum of *m/z* 315.1226 shows the presence of *m/z* 133.06, 121.06, 105.07, and 91.05, indicating that it is a derivative of cinnamic alcohol (Figure S1).

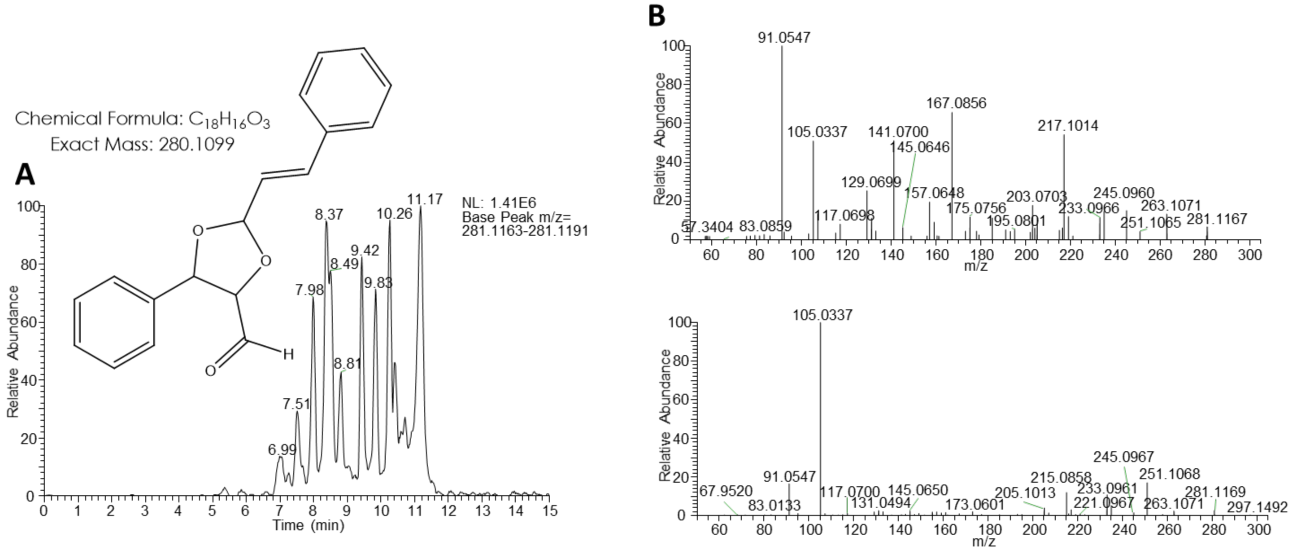

Figure S6. Data could suggest the presence of a dioxolan aldehyde in RHE incubated with cinnamic alcohol.

This is supported by: **A)** An extracted ion chromatogram of *m/z* 281.1177 (5 ppm mass tolerance) in RHE medium incubated with cinnamic alcohol for 24 h revealed an increase in the area of several peaks between RT 6.6-12.0 min over time (2h to 24h); **B)** Two of the formed peaks recorded the accurate MS/MS of *m/z* 281.1169 at retention time 8.44 (upper spectrum) and 9.38 min (lower spectrum), respectively, possibly belonging to the dioxolan cinnamic aldehyde. Adding the theoretical structure to an *in silico* fragmentation software (CFM-ID 4.0[1], available at https://cfmid.wishartlab.com/) revealed several similar fragments are observed such as *m/z* 281.1172 $[M+H]^+$, 263.1066, 251.1066, 175.0753, 131.0491 and 91.0542.

Table S4. Suggested fragmentation structures for the dioxolan derivate identified in the non-target screening.[b]

**Observed *m/z***	**Theoretical *m/z***	**Chemical formula *m/z***	**Chemical loss**	**Structure**	**Smiles**
283.2064	283.1328	$C_{18}H_{19}O_3^+$		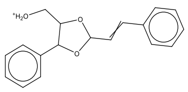	[CH3+]=C1OC(=C=C=C2C=CC=CC2)OC1=C1C=CCCC1
265.1228	265.1223	$C_{18}H_{17}O_2^+$	H_2O	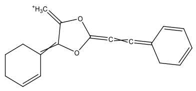	[CH3+]=C1OC(=C=C=C2C=CC=CC2)OC1=C1C=CCCC1
223.1693	223.1117	$C_{16}H_{15}O^+$	$C_2H_4O_2$	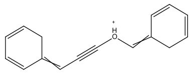	C(#C[OH+]C=C1C=CC=CC1)C=C1C=CC=CC1
177.1638	177.0910	$C_{11}H_{13}O_2^+$	C_7H_6O	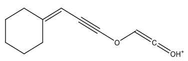	[OH+]=C=COC#CC=C1CCCCC1
133.0649	133.0648	$C_9H_9O^+$	$C_9H_{10}O_2$	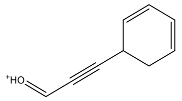	[OH+]=CC#CC1C=CC=CC1
115.0543	115.0542	$C_9H_7^+$	$C_9H_{12}O_3$	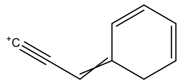	[C+]#CC=C1C=CC=CC1
91.0547	91.0542	$C_7H_7^+$	$C_{11}H_{12}O_3$	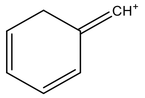	[CH+]=C1C=CC=CC1

[b]Fragmentation structures were predicted with CFM-ID 4.0[1] (available at https://cfmid.wishartlab.com/).

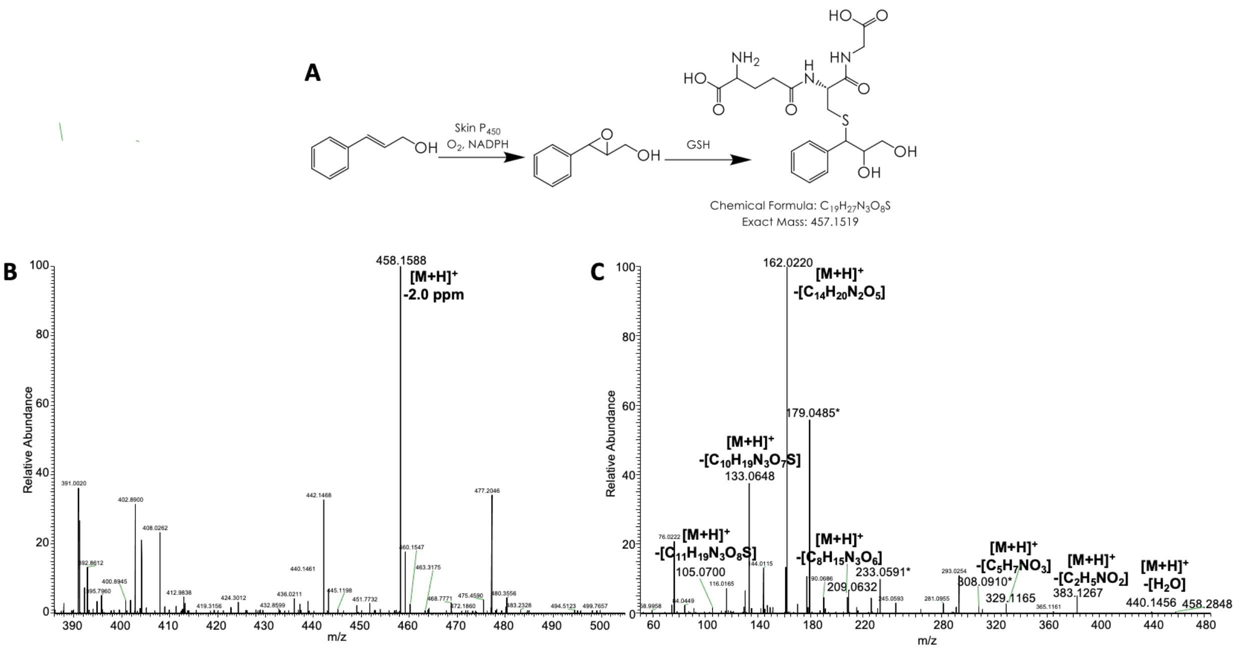

Figure S7. Data suggesting the presence of an epoxy cinnamic alcohol-glutathione adduct

A) Proposed structure and mechanism, adapted from Charpentier et al., for the formation of a glutathione adduct with epoxy cinnamic alcohol[2]. The adduct is formed by an S_N2 reaction of glutathione to epoxy cinnamic alcohol. This formation of this adduct is supported by: **B)** The full MS (RT = 1.11 min) with an addition of $C_9H_{10}N_3O_2$ to glutathione corresponds to a mass accuracy of -2.0 ppm. **C)** The acquired MS/MS spectrum of *m/z* 458.1588 shows the characteristic fragmentation of glutathione indicated in the figure by asterisks (*), and possible losses are detailed.

Table S5. Suggested fragmentation structures for the epoxy cinnamic alcohol-glutathione adduct identified in the non-target screening.[b]

**Observed *m/z***	**Theoretical *m/z***	**Chemical formula *m/z***	**Chemical loss**	**Structure**	**Smiles**
458.1597	458.1588	$C_{19}H_{28}N_3O_8S^+$		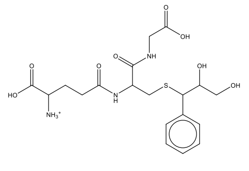	[NH3+]C(C(O)=O)CCC(NC(C(NCC(O)=O)=O)CSC(C(CO)O)c1ccccc1)=O
440.1456	440.1486	$C_{19}H_{26}N_3O_7S^+$	H_2O	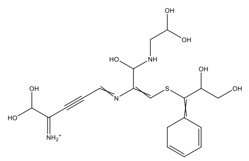	[NH2+]=C(C(O)O)C#CC=NC(C(NCC(O)O)O)=CSC(C(CO)O)=C1C=CC=CC1
383.1267	383.1271	$C_{17}H_{23}N_2O_6S^+$	$C_2H_5NO_2$	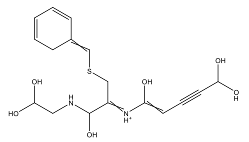	OC([NH+]=C(C(NCC(O)O)O)CSC=C1C=CC=CC1)=CC#CC(O)O
329.1165	329.1166	$C_{14}H_{21}N_2O_5S^+$	$C_5H_7NO_3$	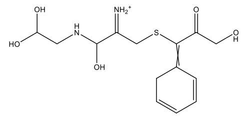	[NH2+]=C(C(NCC(O)O)O)CSC(C(CO)=O)=C1C=CC=CC1
209.0632	209.0631	$C_{11}H_{13}O_2S^+$	$C_8H_{15}N_3O_6$	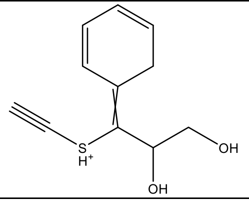	C#C[SH+]C(C(CO)O)=C1C=CC=CC1
162.0220	162.0219	$C_5H_8NO_3S^+$	$C_{14}H_{20}N_2O_5$	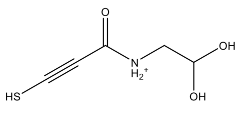	O=C([NH2+]CC(O)O)C#CS
133.0648	133.0648	$C_9H_9O^+$	$C_{10}H_{19}N_3O_7S$	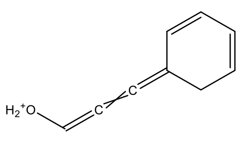	[OH2+]C=C=C=C1C=CC=CC1

105.0700	105.0699	$C_8H_9^+$	$C_{11}H_{19}N_3O_8S$	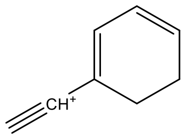	C#[CH+]C1=CC=CCC1
**Diagnostic fragments of GSH in positive ESI mode**					
308.0910	308.0911	$C_{10}H_{18}N_3O_6S^+$	$C_9H_{10}O_2$	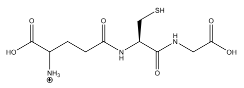	O=C(N[C@@H](CS)C(NCC(O)=O)=O)CCC([NH3+])C(O)=O
233.0591	233.0591	$C_8H_{13}N_2O_4S^+$	$C_{11}H_{15}NO_4$	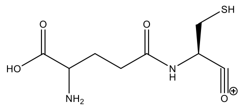	O=C(N[C@@H](CS)C#[O+])CCC(N)C(O)=O
179.0485	179.0485	$C_5H_{11}N_2O_2S^+$	$C_{14}H_{17}NO_6$	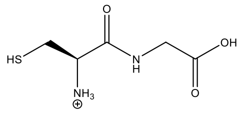	O=C(NCC(O)=O)[C@H](C[SH+])[NH3]

[b]Fragmentation structures were predicted with CFM-ID 4.0[1] (available at https://cfmid.wishartlab.com/).

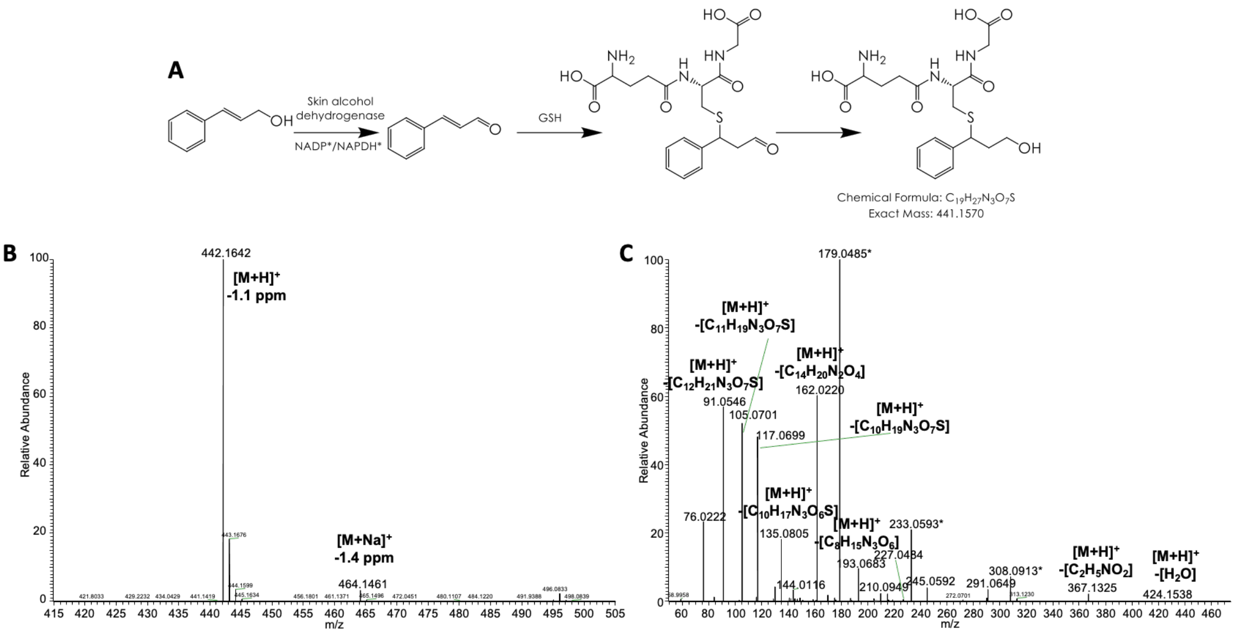

Figure S8. Data suggesting the presence of a cinnamic aldehyde-glutathione adduct

A) Proposed structure and mechanism, adapted from Charpentier et al., for the formation of a glutathione adduct with cinnamic aldehyde[2]. The first step is a Michael addition of glutathione to cinnamic aldehyde followed by a reduction of the aldehyde to an alcohol. This formation of this adduct is supported by: **B)** The full MS (RT = 2.15 min) with an addition of $C_{10}H_{10}O$ to glutathione corresponds to a mass accuracy of -1.1 ppm. **C)** The acquired MS/MS spectrum of *m/z* 442.1642 shows the characteristic fragmentation of glutathione, indicated in the figure by asterisks (*), and possible losses are detailed.

Table S6. Suggested fragmentation structures for the glutathione-cinnamic aldehyde adduct identified in the non-target screening.[b]

Observed *m/z*	Theoretical *m/z*	Chemical formula *m/z*	Chemical loss	Structure	Smiles
442.1642	442.1647	$C_{19}H_{28}N_3O_7S^+$		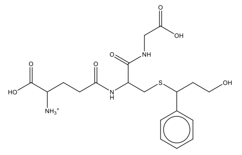	[NH3+]C(C(O)=O)CCC(NC(C(NCC(O)=O)=O)CSC(c1ccccc1)CCO)=O
424.1538	424.1537	$C_{19}H_{26}N_3O_6S^+$	H_2O	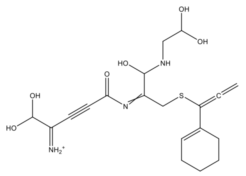	C=C=C(C1=CCCCC1)SCC(C(NCC(O)O)O)=NC(C#CC(C(O)O)=[NH2+])=O
367.1325	367.1322	$C_{17}H_{23}N_2O_5S^+$	$C_2H_5NO_2$	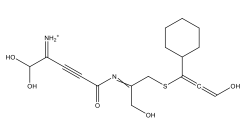	[NH2+]=C(C(O)O)C#CC(N=C(CSC(C1CCCCC1)=C=CO)CO)=O
193.0683	193.0682	$C_{11}H_{13}OS^+$	$C_8H_{15}N_3O_6$	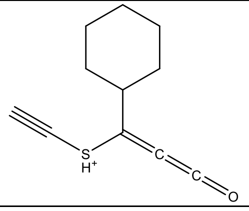	C#C[SH+]C(C1CCCCC1)=C=C=O
162.0220	162.0219	$C_5H_8NO_3S^+$	$C_{14}H_{20}N_2O_4$	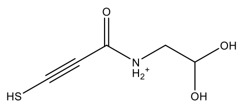	O=C([NH2+]CC(O)O)C#CS
135.0805	135.0804	$C_9H_{11}O^+$	$C_{10}H_{17}N_3O_6S$	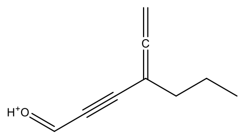	C=C=C(CCC)C#CC=[OH+]

117.0699	117.0699	$C_9H_9^+$	$C_{10}H_{19}N_3O_7S$	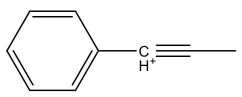	CC#[CH+]C1=CC=CC=C1
105.0701	105.0699	$C_8H_9^+$	$C_{11}H_{19}N_3O_7S$	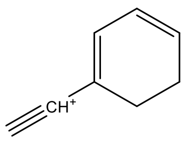	C#[CH+]C1=CC=CCC1
91.0546	91.0542	$C_7H_7^+$	$C_{12}H_{21}N_3O_7S$	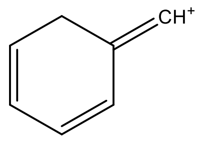	[CH+]=C1C=CC=CC1
**Diagnostic fragments of GSH in positive ESI mode**					
308.0913	308.0911	$C_{10}H_{18}N_3O_6S^+$	$C_9H_{10}O$	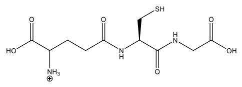	O=C(N[C@@H](CS)C(NCC(O)=O)=O)CCC([NH3+])C(O)=O
233.0593	233.0591	$C_8H_{13}N_2O_4S^+$	$C_{11}H_{15}NO_3$	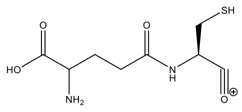	O=C(N[C@@H](CS)C#[O+])CCC(N)C(O)=O
179.0485	179.0485	$C_5H_{11}N_2O_2S^+$	$C_{14}H_{17}NO_5$	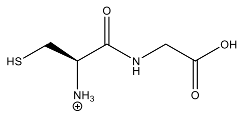	O=C(NCC(O)=O)[C@H](C[SH+])[NH3]

[b]Fragmentation structures were predicted with CFM-ID 4.0[1] (available at https://cfmid.wishartlab.com/).

Murine local lymph node assay

Table S7. Results from the murine local lymph node assay (LLNA)[a]: Sensitization experiments of the two pOH-cinnamic compounds and the control cinnamic aldehyde.

Compd and test concentration (% w/v)	Compd and test concentration (M)	[^{3}H]thymidine incorporation (dpm/lymph node)	SI	EC$_3$ value % w/v	EC$_3$ value M	Classification
Cinnamic aldehyde				0.75	0.057	Strong
Control		329				
0.10	7.5×10^{-3}	381	1.2			
0.99	75×10^{-3}	1211	3.7			
3.30	0.25	2075	6.3			
9.90	0.75	3967	12			
19.80	1.5	4652	14			
pOH-Cinnamic aldehyde				6.11	0.42	Moderate
Control		329				
3	6.8×10^{-3}	379	1.2			
6	68×10^{-3}	468	1.4			
12	0.20	469	1.4			
18	0.61	1194	3.6			
27	1.2	1322	4.0			
pOH-Cinnamic alcohol				n/a	n/a	Non-sensitizer
Control		180				
3	0.20	165	0.9			
6	0.40	225	1.2			
12	0.80	370	2.3			
18	1.2	326	1.8			
27	1.8	330	1.8			

[a]Groups of mice were treated with test substance in 5 different concentrations, on the dorsum of both ears for 3 consecutive days. Sham treated control animals received vehicle alone. On day 5, all mice were injected intravenously with PBS (250 µl) containing 20 µCi of [^{3}H]-methylthymidine. After 5 h the mice were sacrificed, the draining lymph nodes were excised and pooled for each group, single cell suspensions of lymph-node cells were prepared, and the thymidine incorporation into DNA was measured by *β*-scintillation counting. The increase in thymidine incorporation relative to vehicle-treated controls was derived for each experimental group and recorded as stimulation index[3]. The EC$_3$ values (the estimated concentration required to induce an SI of 3) were calculated using linear interpolation. The sensitizing potency was classified to the following: <0.1%, extreme; ≥0.1 to <1%, strong; ≥1 to <10%, moderate; and ≥10%, weak[4]. [b]Molarity was calculated based on the molecular weight of cinnamic alcohol.

References

(1) Wang, F.; Liigand, J.; Tian, S.; Arndt, D.; Greiner, R.; Wishart, D. S. CFM-ID 4.0: More Accurate ESI-MS/MS Spectral Prediction and Compound Identification. *Anal. Chem.* **2021**, *93* (34), 11692–11700. https://doi.org/10.1021/acs.analchem.1c01465.

(2) Charpentier, J.; Emter, R.; Koch, H.; Lelièvre, D.; Pannecoucke, X.; Couve-Bonnaire, S.; Natsch, A.; Bombrun, A. Effect of Fluorination on Skin Sensitization Potential and Fragrant Properties of Cinnamyl Compounds. *Chemistry & Biodiversity* **2018**, *15* (4), e1800013. https://doi.org/10.1002/cbdv.201800013.

(3) Gerberick, F.; Aleksic, M.; Basketter, D.; Casati, S.; Karlberg, A.-T.; Kern, P.; Kimber, I.; Lepoittevin, J. P.; Natsch, A.; Ovigne, J. M.; Rovida, C.; Sakaguchi, H.; Schultz, T. Chemical Reactivity Measurement and the Predictive Identification of Skin Sensitisers: The Report and Recommendations of ECVAM Workshop 64a. *Altern Lab Anim* **2008**, *36* (2), 215–242. https://doi.org/10.1177/026119290803600210.

(4) Kimber, I.; Basketter, D. A.; Butler, M.; Gamer, A.; Garrigue, J.-L.; Gerberick, G. F.; Newsome, C.; Steiling, W.; Vohr, H.-W. Classification of Contact Allergens According to Potency: Proposals. *Food and Chemical Toxicology* **2003**, *41* (12), 1799–1809. https://doi.org/10.1016/S0278-6915(03)00223-0.
